# Molecular and microenvironmental drivers of malignant transformation from oral potentially malignant disorders to oral squamous cell carcinoma

**DOI:** 10.3389/fonc.2026.1856931

**Published:** 2026-06-03

**Authors:** Vasundra V., Shajitha R., Sundaresan S., Magesh R.

**Affiliations:** 1 Department of Biotechnology, Faculty of Biomedical Sciences & Technology, Sri Ramachandra Institute of Higher Education and Research (DU), Chennai, India; 2 Division of Medical Research, SRM MCH and RC, SRM IST, Kattankulathur, Tamil Nadu, India

**Keywords:** biomarkers, healthcare, malignant transformation, multi-omics, oral potentially malignant disorders, oral squamous cell carcinoma, precision oncology, tumor microenvironment

## Abstract

Oral potentially malignant disorders (OPMDs) comprise a diverse group of oral mucosal lesions with a variable but significant risk of progression to oral squamous cell carcinoma (OSCC). However, predicting malignant transformation remains a major clinical challenge due to the limited reliability of conventional histopathological grading and the inherent biological heterogeneity of these lesions. Increasing evidence indicates that OPMD progression is a dynamic, multistep process driven by the convergence of molecular alterations and microenvironmental remodeling. This review synthesizes current knowledge on the key mechanisms underlying OPMD-to-OSCC transformation, focusing on the interplay between genomic instability, epigenetic dysregulation, chronic inflammation, and tumor microenvironment evolution. Dysregulation of critical signaling pathways, including TGF-β, PI3K/AKT, MAPK, Wnt/β-catenin, and NF-κB, contributes to epithelial plasticity, immune evasion, fibrosis, and sustained oncogenic signaling. Special attention is given to oral submucous fibrosis as a high-risk model, highlighting the role of extracellular matrix stiffening and mechanotransduction pathways such as YAP/TAZ in promoting malignant progression. The limitations of morphology-based diagnostics underscore the need for molecularly informed risk assessment in modern healthcare. Emerging biomarkers including genetic, epigenetic, transcriptomic, proteomic, metabolomic, and exosomal signatures offer promising tools for early detection and stratification of high-risk lesions. Furthermore, integration of multi-omics data with artificial intelligence based predictive models holds significant potential for advancing precision oncology in oral cancer. Collectively, this review supports an evolutionary shift in understanding OPMDs as dynamic and evolving biological systems rather than static precursor lesions. Elucidating the molecular and microenvironmental convergence driving malignant transformation provides a foundation for improved early detection, risk prediction, and development of targeted preventive and therapeutic strategies in OSCC.

## 1 Introduction

Oral squamous cell carcinoma (OSCC) is the most common form of oral cancer and remains a major global health challenge due to its high incidence, aggressive progression, and poor five-year survival rates (3). The burden is particularly high in South and Southeast Asia, largely driven by risk factors such as tobacco use, areca nut consumption, alcohol intake, and poor oral hygiene. Despite advances in surgery, radiotherapy, and chemotherapy, survival outcomes have improved only marginally, primarily due to late-stage diagnosis (1, 2).

OSCC often arises from oral potentially malignant disorders (OPMDs), including leukoplakia, erythroplakia, oral submucous fibrosis (OSMF), and oral lichen planus. These lesions exhibit heterogeneous clinical and biological behavior, with malignant transformation rates varying according to lesion type, geographic region, and associated risk factors (4). Conventional diagnostic approaches based on clinical examination and histopathological grading remain limited, as lesions with similar histology can demonstrate markedly different clinical outcomes (3). This highlights the inadequacy of morphology-based assessment in predicting disease progression.

Emerging evidence indicates that OPMD progression to OSCC is a dynamic, multistep process driven by cumulative genetic mutations, epigenetic alterations, metabolic reprogramming, and chronic inflammation (5, 6). Dysregulation of key oncogenic pathways, including TGF-β, PI3K/AKT, Wnt/β-catenin, and NF-κB, contributes to genomic instability, uncontrolled proliferation, and malignant transformation. Importantly, these molecular changes often precede visible histological alterations, offering opportunities for early risk detection (7).

The tumor microenvironment plays a central role in this transition. Chronic inflammation, oxidative stress, hypoxia, and immune dysregulation create a pro-tumorigenic niche that promotes epithelial plasticity, angiogenesis, and immune evasion. In conditions such as OSMF, excessive extracellular matrix deposition increases tissue stiffness and activates mechanotransduction pathways that further drive tumor progression (8, 9).

The entire field requires systems-level perspective adoption to achieve its advancement. Systems biology uses multiple types of biological data, including genomics, transcriptomics, proteomics, epigenomics, and metabolomics, to model biological systems that lead to disease advancement (10, 11). The method combines spatial pathology and longitudinal sampling with computational modeling to create an advanced approach which examines the combined effects of molecular changes and environmental factors and time progression on clinical results (12). The use of these integrated methods will transform current risk assessment processes because they will enable detection of high-risk lesions at an early stage and help identify treatment points, which will manage cancer development before it reaches an advanced stage (10).

### 1.1 Methods for literature selection

This narrative review was conducted through a comprehensive literature search using electronic databases including PubMed, Scopus, Web of Science, and Google Scholar. Relevant literature published in English up to 2025 was retrieved to identify studies related to oral potentially malignant disorders (OPMDs), oral submucous fibrosis (OSMF), oral squamous cell carcinoma (OSCC), tumor microenvironment remodeling, signaling pathways, biomarkers, multi-omics technologies, and artificial intelligence–based diagnostic approaches.

The search strategy included combinations of keywords such as “oral potentially malignant disorders,” “OSMF,” “OSCC,” “malignant transformation,” “tumor microenvironment,” “fibrosis,” “epithelial–mesenchymal transition,” “signaling pathways,” “multi-omics,” “exosomes,” and “artificial intelligence.” Original research articles, systematic reviews, meta-analyses, and clinically relevant experimental studies were included based on scientific relevance and contribution to the objectives of the review. Non-English articles, duplicate studies, conference abstracts without full text, and studies lacking sufficient methodological clarity or relevance were excluded. Priority was given to recent studies and high-impact publications that provided mechanistic, translational, and clinical insights into OPMD-to-OSCC progression.

### 1.2 OSMF as a high-risk pre-cancer model

Oral submucous fibrosis (OSMF) is a chronic, progressive, and potentially malignant disorder of the oral mucosa. It is marked by an excessive development of extracellular matrix components and the gradual fibrosis of oral tissues (13). This condition is common in South and Southeast Asia since many people there chew areca nut, betel quid, and tobacco. Arecoline, an alkaloid found in areca nuts, plays a significant role in the development of oral submucous fibrosis (OSMF). It stimulates fibroblast proliferation, enhances collagen synthesis, and disrupts the equilibrium between the formation and degradation of the extracellular matrix (14).

The increasing fibrosis in OSMF establishes a unique microenvironment characterized by epithelial atrophy, reduced blood circulation, chronic inflammation, and diminished oxygen levels inside the tissue (15). These changes make the genome less stable, cause epithelial-mesenchymal transition (EMT), and activate oncogenic signaling pathways that facilitate cellular transformation into malignant states. Epidemiological studies indicate that the rate of malignant transformation in OSMF ranges from 7% to 13%, which shows how important it is as a high-risk precancerous syndrome (13, 16). OSMF is a significant biological model for understanding the molecular mechanisms linking chronic fibrosis to epithelial carcinogenesis because of its fibrosis-driven pathogenesis and well-defined progression toward OSCC. Moreover, OSMF uniquely integrates fibrosis, chronic inflammation, hypoxia, and extracellular matrix stiffening within a single disease model, making it particularly valuable for studying the molecular and microenvironmental drivers of oral carcinogenesis. Accordingly, OSMF has been given particular emphasis in this review as a representative fibrosis-associated model of oral carcinogenesis, providing insights into extracellular matrix remodeling, mechanotransduction, and fibrosis-associated malignant transformation. Nevertheless, other major OPMDs, including leukoplakia and oral lichen planus, are also discussed in relation to the shared molecular and microenvironmental mechanisms underlying OSCC progression.

### 1.3 Conceptual framework: fibrosis-to-carcinoma transition

The transition from oral submucous fibrosis (OSMF) to oral squamous cell carcinoma (OSCC) is defined as a fibrosis-induced carcinogenic route, wherein continuous stromal remodeling and epithelial modifications together facilitate malignant transformation (17). Long-term exposure to areca nut alkaloids creates oxidative stress that maintains fibrogenic signaling pathways, particularly those involving transforming growth factor-β (TGF-β) (18). This excessive production of extracellular matrix (ECM), which activates myofibroblasts and slowly causes tissue fibrosis to grow over time. As the fibrotic matrix grows, it changes the structure and mechanical properties of the oral mucosa. This makes the tissue tougher, stops blood vessels from growing, and kills epithelial cells. When the microenvironment changes, epithelial cells are more likely to be genetically unstable and act abnormally (19).

Multiple oncogenic signaling pathways, including TGF-β, PI3K/AKT, MAPK, and Wnt/β-catenin, are actively involved in the progression from oral submucous fibrosis (OSMF) to oral squamous cell carcinoma (OSCC) (20). These pathways collectively regulate cell proliferation, epithelial–mesenchymal transition, and immune evasion and are further amplified by chronic inflammation and hypoxia. The interplay between epithelial genetic alterations and the stromal microenvironment drives malignant transformation, highlighting the importance of understanding fibrosis-to-carcinoma transition for early detection and targeted prevention (21).

The article presents an in-depth review of the current scientific knowledge regarding the molecular and microenvironmental mechanisms that promote the progression of oral potentially malignant disorders (OPMDs) to oral squamous cell carcinoma (OSCC) (14). The review produces data from systems-level studies, highlighting prominent oncogenic signaling pathways, innovative biomarkers, and potential therapeutic targets linked to malignant transformation (22). This perspective emphasizes the need to discover OPMDs as dynamic biological systems, enabling the development of precise surveillance strategies and individualized chemopreventive medicines aimed at improving clinical outcomes in oral cancer.

## 2 Epidemiology and clinical burden

### 2.1 Global and Indian scenario with malignant transformation rates

Public health professionals consider oral potentially malignant disorders (OPMDs) as an important health threat because these conditions lead to oral squamous cell carcinoma (OSCC), which represents more than 90% of all oral cancers that exist globally (23). Epidemiological studies indicate that OPMDs are measurable indicators of exposure to carcinogenic substances, social health factors, and lack of access to preventive dental services (3). The global distribution of OPMDs shows significant regional disparities that arise from differences in people’s daily routines, cultural customs, economic circumstances, and their capacity to receive medical treatment. Effective cancer prevention strategies necessitate that researchers investigate the epidemiology of OPMD, as the information enables the identification of individuals requiring specialized monitoring (24).

**Figure 1:**
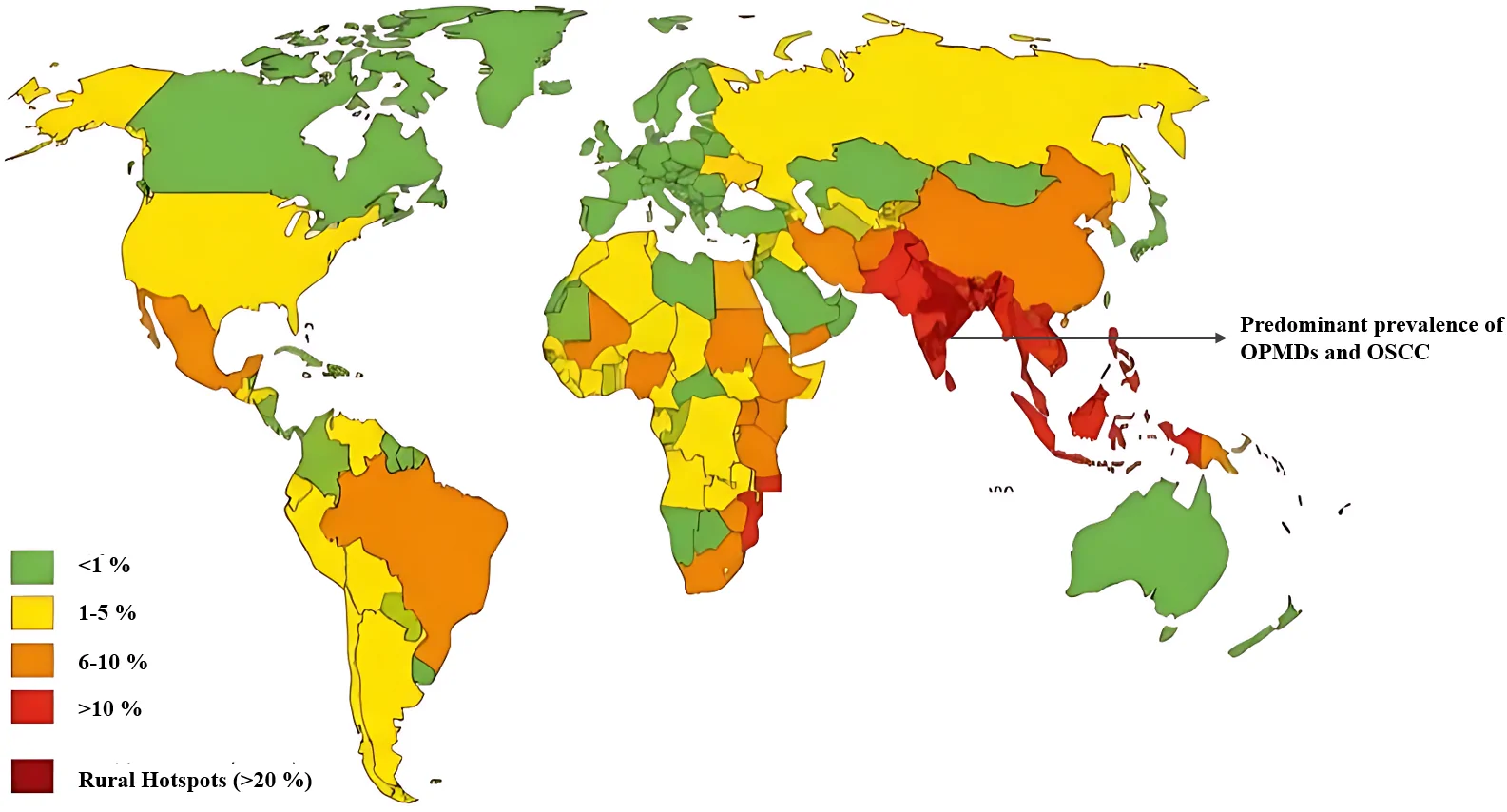
Illustrates the global and regional distribution of oral potentially malignant disorders and their relationship with oral squamous cell carcinoma incidence.

The global OPMD prevalence rate ranges from 1% to 5%, yet this figure hides significant differences between different groups of people and different types of lesions (23). The presence of various diagnostic criteria together with examiner expertise and age distribution and Participant behavioral risk factors leads to different prevalence rates according to large epidemiological studies and meta-analyses (**Figure 1**) (25). Individuals in regions with minimal tobacco and areca nut consumption exhibit a reduced incidence of OPMD, as dental examinations facilitate the early detection of the condition (26). The use of smokeless tobacco and betel quid together with areca nut has established a pattern in several countries, which leads to increased OPMD rates and more severe disease manifestations (27).

Oral leukoplakia is recognized globally as the most prevalent potentially malignant condition of the oral cavity (28). The medical definition of leukoplakia describes it as a white oral mucosa plaque which medical professionals cannot identify as any other known condition. The occurrence of this disease strongly connects with tobacco smoking, including both cigarette and bidi smoking and all forms of smokeless tobacco (24). The condition of leukoplakia develops in non-smokers who experience continuous mechanical damage to their bodies and drink alcohol and have genetic factors which make them vulnerable to the disease (28, 29).

Erythroplakia is a significant oral potentially malignant disorder due to its infrequent occurrence and markedly elevated risk of cancer development (30). Erythroplakia presents as a red velvety oral mucosa lesion that has clear boundaries and affects people across the globe with a prevalence rate of 0.17%. Erythroplakia is more common in older adults, and it is more strongly linked to drinking alcohol and smoking tobacco than it is in younger people (27).

Oral submucous fibrosis is a specific type of OPMD that is only found in South and Southeast Asia (26). It has its own unique pattern of spread. OSMF develops as a progressive fibrotic condition that leads to mouth opening restrictions and a burning feeling, which affects the mouth lining and results in epithelial atrophy and dysplasia (30, 31). The global prevalence of OSMF varies, with estimates suggesting it impacts around 5% of individuals in high-risk populations where areca nut chewing is common. Eating areca nuts, whether alone or with betel quid, is a Group 1 carcinogen and the main cause of OSMF. Epidemiological studies demonstrate that the chance of acquiring OSMF increases with greater exposure to areca nut, and persons who begin chewing at a younger age experience an earlier onset of the illness (31).

India is currently one of the countries with the most OPMD cases because its big population and existing economic inequities make it more likely to have several risk factors. The OPMD prevalence studies conducted through population-based research methods show results between 13% and 14%, which exceeds the global average (31, 32). The high prevalence rate shows the common usage of smokeless tobacco products and betel quid and gutkha and areca nut products among different population groups. India demonstrates a unique pattern of lesion distribution, which shows that most cases involve leukoplakia and OSMF, while erythroplakia and oral lichen planus follow behind.

The research established that OPMD conditions show different rates of occurrence across India because local cultural practices and economic factors and the existence of urban and rural areas create different patterns of disease distribution. The study found that leukoplakia affected about 8.6% of participants, OSMF affected 4.8%, and erythroplakia occurred in 2.8% of cases (**Figure 1**) (33). The study found that different states and districts in India showed substantial variations in results (31). The southern and northeastern areas of India experience more OSMF cases because of their high areca nut usage, while northern areas show higher leukoplakia cases that result from tobacco consumption. The existing geographical differences in disease patterns, because of national average data, require specialized disease tracking systems for each region (33, 34).

Rural communities in India experience a particularly high burden of oral potentially malignant disorders, with prevalence rates exceeding 20% in certain populations. The enormous number of people who smoke tobacco and eat areca nuts, as well as society’s support of these items, makes this sickness load much worse (35). People who live in rural locations have a hard time receiving healthcare. For example, they can’t see dentists or get screened, which makes it tougher to discover tumors that might be cancerous soon. Additional variables that raise the likelihood of lesions forming and lasting longer include being exposed to certain jobs, lacking sufficient nutrients, and neglecting your teeth and gums (35, 36).

The age and gender patterns of OPMD cases reveal significant public health insights regarding disease distribution. OPMD shows a common occurrence among people who reach their middle age and advanced age, but OSMF cases now affect younger people because they started using areca nuts at earlier ages. More men than women have historically higher rates of tobacco and alcohol use, which results in increased health problems. The social practices of some areas have led to better health outcomes for women because they now use tobacco products at the same rate as men.

OPMDs are a major public health concern, especially in regions with high-risk habits and limited resources. Their prevalence varies by lesion type, geography, and population groups. In India, the high burden of OPMDs contributes to increased OSCC cases, highlighting the need for early detection, prevention programs, and tobacco cessation. Epidemiological studies support risk assessment and help guide effective oral cancer prevention strategies.

### 2.2 Clinical heterogeneity and prognostic challenges

Oral potentially malignant disorders (OPMDs) and oral squamous cell carcinoma (OSCC) demonstrate significant clinical variability, hindering early identification and risk assessment. Oral potentially malignant disorders, including leukoplakia, erythroplakia, oral submucous fibrosis, and oral lichen planus, vary in their presentation and risk of malignancy. Some lesions remain stable or regress, while others advance to OSCC due to genetic, environmental, and behavioral influences (4).

OSCC exhibits variety in tumor behavior, metastatic potential, treatment response, and patient outcomes. Epithelial dysplasia, chronic inflammation, and molecular changes influence the transition from OPMDs to OSCC; however, histopathological grading alone fails to provide a precise prognosis. These issues underscore the necessity for integrated methodologies that amalgamate clinical characteristics, molecular biomarkers, and prolonged monitoring to enhance risk assessment and facilitate early intervention (37).

### 2.3 Oral squamous cell carcinoma: incidence and mortality rates

Oral squamous cell carcinoma (OSCC) accounts for approximately 90% of oral cancers and remains a major global public health challenge. According to GLOBOCAN 2020, around 377,700 new cases and 177,757 deaths were reported worldwide, reflecting its substantial impact on cancer-related morbidity and mortality. The global burden of OSCC shows marked geographic variation, largely influenced by lifestyle factors, socioeconomic conditions, and access to healthcare (38) (**Table 1**).

**Table 1:** Global epidemiological overview of OSCC based on GLOBOCAN data and associated risk factors (41).

Parameter	Details
Disease Type	Oral Squamous Cell Carcinoma (OSCC) (~90% of oral cancers)
Global Incidence (GLOBOCAN 2020)	~377,700 new cases/year
Global Mortality (GLOBOCAN 2020)	~177,757 deaths/year
Projected Incidence (GLOBOCAN 2040)	~40% increase in cases worldwide
Geographic Distribution	Highest in South and Southeast Asia
High-Burden Country	India (~130,000–140,000 new cases/year)
Major Risk Factors	Tobacco, alcohol, areca nut/betel quid
Emerging Risk Factor	HPV (subset of cases, especially younger population)
Age Trend	Traditionally older adults; increasing in younger groups
Stage at Diagnosis	Majority detected at advanced stages
5-Year Survival (Early Stage)	~70–80%
5-Year Survival (Advanced Stage)	<30%
Key Challenges	Late diagnosis, limited screening, poor healthcare access
Prevention Strategies	Tobacco cessation, areca nut control, early detection programs
Clinical Relevance	Strong association with OPMDs; early intervention reduces OSCC risk

The highest incidence rates are observed in South and Southeast Asia, particularly in India, where widespread use of tobacco, betel quid, and areca nut significantly increases disease risk. India alone contributes approximately 130,000 new cases annually (39). Limited access to screening and healthcare services in many regions leads to delayed diagnosis and poor clinical outcomes.

Despite advances in treatment, OSCC continues to have low survival rates, primarily due to late-stage detection. Early-stage disease is associated with favorable five-year survival rates of 70–80%, whereas advanced-stage OSCC often shows survival rates below 30%. A large proportion of patients present with advanced or metastatic disease, limiting therapeutic options (39, 40).

Future projections indicate a potential 40% increase in global OSCC incidence by 2040, particularly affecting low- and middle-income countries. While tobacco and alcohol remain the primary risk factors, emerging evidence highlights the role of human papillomavirus (HPV) in a subset of cases (40). Importantly, the strong association between OSCC and oral potentially malignant disorders (OPMDs) underscores the importance of early detection and intervention. Implementation of screening programs, risk factor control, and biomarker-based approaches is essential for reducing disease burden and improving clinical outcomes.

## 3 Etiological and environmental drivers of OSMF

### 3.1 Areca nut and alkaloid-induced fibrogenesis

**Figure 2:**
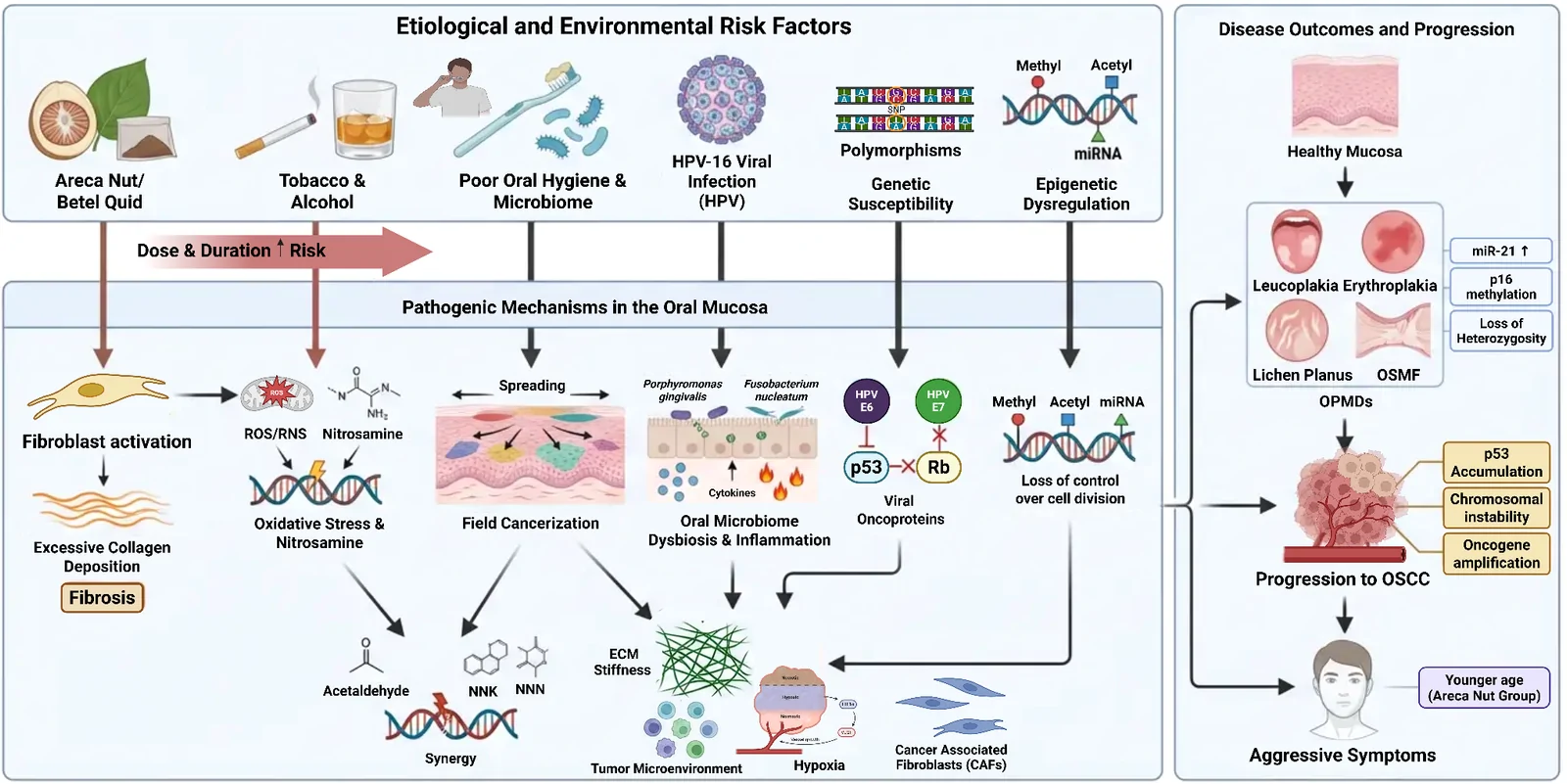
The figure illustrates the stepwise progression from environmental exposures such as areca nut, tobacco, alcohol, and microbial dysbiosis to oxidative stress, chronic inflammation, epigenetic alterations, and activation of oncogenic pathways that collectively contribute to OSMF development and malignant transformation into OSCC.

People in South and Southeast Asia engage in betel quid and areca nut chewing as traditional customs, which create the highest risk for OSCC in these territories. The International Agency for Research on Cancer (IARC) classifies areca nut and betel quid, with or without tobacco, as Group 1 carcinogens (42). Areca nuts contain alkaloids such as arecoline, which stimulate fibroblast proliferation and collagen synthesis to produce excessive extracellular matrix deposition that leads to progressive fibrosis (**Figure 2**) (43).

The pathological processes of oral submucous fibrosis (OSMF) create a high-risk OPMD, which shows mucosal rigidity and epithelial atrophy and reduced vascularity (44). The OSMF microenvironment creates fibrotic conditions which produce mechanical stiffness and hypoxia that activate oncogenic signaling pathways while hindering epithelial cell differentiation. The DNA damage and genomic instability of cells occur because nitrosamines and ROS are produced during areca nut metabolism (17, 44).

Research studies conducted on entire populations, together with meta-analyses, show that betel quid chewing creates a strong dose-dependent relationship with OSCC, which increases the risk of transformation through more frequent chewing and longer chewing duration and earlier initiation of the habit (45). The OSCC cases which develop from areca nut use show that patients reach their diagnosis at a younger age and display more aggressive clinical symptoms (45, 46).

### 3.2 Oxidative stress and nitrosamine exposure

Oxidative stress is crucial in the start and growth of oral squamous cell carcinoma (OSCC) and oral submucous fibrosis (OSMF) (47). Long-term use of areca nut, tobacco, and betel quid creates reactive oxygen species (ROS) and reactive nitrogen species (RNS) in the mouth. These reactive species cause oxidative damage to DNA, lipids, and proteins, which makes the genome unstable and messes up important cellular pathways that control cell growth and death (47, 48).

Moreover, the metabolism of tobacco and areca nut generates carcinogenic nitrosamines, such as N-nitrosonornicotine (NNN) and 4-(methylnitrosamino)-1-(3-pyridyl)-1-butanone (NNK) (**Figure 2**). These substances promote the formation of DNA adducts and mutations in oral epithelial cells, increasing their susceptibility to carcinogenesis (49). Chronic exposure to oxidative stress and nitrosamines creates a pro-tumorigenic milieu marked by epithelial dysplasia, genomic instability, and a gradual progression towards malignancy.

### 3.3 Tobacco and alcohol synergy

Individuals can most effectively prevent oral squamous cell cancer by entirely ceasing tobacco consumption. Both smoked and smokeless tobacco include numerous carcinogenic compounds that harm the DNA of oral cells. There are three primary ways that smoking tobacco might harm your DNA (50). They include polycyclic aromatic hydrocarbons (PAHs), nitrosamines that are only found in tobacco, and reactive oxygen species (ROS) (**Figure 2**). These chemical injuries often affect important tumor suppressor genes, such as TP53. This process complicates the cessation of cellular division, diminishes the efficacy of apoptosis, and permits the survival of genetically compromised clones.

Chronic tobacco exposure leads to two outcomes, which include random mutation and clonal evolution, through its impact on oral epithelium cells. Cancer cells lacking appropriate DNA damage response mechanisms exhibit enhanced survival when subjected to carcinogenic treatment (51). This leads to more of them living and growing until they control the whole area. The process explains field cancerization, which describes how genetically modified cells spread through large regions of normal-looking mucosal tissue (50).

Smokeless tobacco products, which include gutkha and snuff and chewing tobacco, create carcinogenic risks that match the dangers of smoking (52). These products expose the oral mucosa to prolonged contact with high concentrations of nitrosamines, particularly N-nitrosonornicotine (NNN) and 4-(methylnitrosamino)-1-(3-pyridyl)-1-butanone (NNK). The epidemiological meta-analyses show that smokeless tobacco use creates a strong connection, which depends on both dose and duration, between its effects on health.

#### 3.3.1 Alcohol

The emergence of OSCC is contingent upon alcohol consumption, which serves as a significant co-carcinogen (53). Ethanol does not cause mutations directly, but its main metabolite, acetaldehyde, shows strong genetic damage effects. Acetaldehyde produces permanent DNA-binding compounds which disrupt DNA repair processes and lead to chromosomal abnormalities, including sister chromatid exchanges (54). The oral cavity shows increased acetaldehyde accumulation because specific bacteria break down acetaldehyde, and certain people have a decreased ability to process the compound.

Ethanol acts as a solvent, which makes oral mucosa more permeable while allowing tobacco-related cancer-causing substances to enter the body. Alcohol consumption activates cytochrome P450 enzymes, especially CYP2E1, which transforms cancer-causing substances into their active form while producing reactive oxygen species. The combination of tobacco and alcohol use creates a biochemical interaction that results in OSCC risk, which increases more than the sum of risks from each substance used separately (53, 54).

### 3.4 Poor oral hygiene, periodontal disease, and microbiome influences

OSCC risk increases because people with poor oral hygiene and chronic periodontal disease sustain permanent inflammation, which leads to bacterial imbalance. The process of dental plaque accumulation, together with periodontitis, results in continuous inflammation, which produces cytokines and creates oxidative stress while the body replaces damaged epithelial tissue. Certain oral bacteria can convert ethanol into acetaldehyde, and they can transform nitrates into nitrosamines, which produce local genotoxic compounds (**Figure 2**) (55).

The pathogenic species Porphyromonas gingivalis and Fusobacterium nucleatum cause oral cancer development because they can change how the immune system works and they break down epithelial connections while their invasive capabilities develop (55, 56). These microbes can create cancer-causing signaling mechanisms while they hinder anti-cancer defense systems and help their immune system protection. Poor oral hygiene together with tooth loss increases the risk of developing oral cancer, according to clinical studies which adjusted their results for tobacco and alcohol consumption (55–57).

Beyond direct carcinogenic effects, microbiome dysbiosis also influences host immune responses and tumor microenvironment remodeling. Pathogenic oral microbes can modulate inflammatory cytokine production, alter immune cell recruitment, and promote chronic immunosuppressive signaling, thereby contributing to epithelial transformation and tumor progression (4, 58).

### 3.5 Viral carcinogenesis

High-risk human papillomaviruses (HPVs), particularly HPV-16, create a biologically separate version of head and neck squamous cell carcinomas, which mostly affects the oropharynx while it also impacts the oral cavity in smaller amounts (**Figure 2**). The epidemiological patterns together with the molecular characteristics and clinical manifestations of HPV-related OSCC differ from those of chemically induced tumors (59).

The viral oncoproteins E6 and E7 serve as the main agents which drive HPV-related cancer development. The E6 protein establishes a partnership with E6AP, the ubiquitin ligase to direct the p53 tumor suppressor protein toward degradation through ubiquitination and subsequent proteasomal destruction (60). The absence of p53 function creates a major genomic monitoring system failure, which permits DNA-damaged cells to survive and keep growing.

The E7 protein alters the retinoblastoma (Rb) tumor suppressor pathway. When everything is working regularly, Rb protein stops the cell cycle from progressing forward by attaching to E2F transcription factors (61). When E7 protein attaches to Rb, it stops Rb from executing its job. This allows E2F to go and slows down the S-phase. When cells are forced to divide and their DNA damage protection systems don’t work well, it makes the DNA more unstable and cancer cells grow. Cancers associated with HPV exhibit distinct biological characteristics, including the overexpression of p16, and also exhibit varied responses to treatment (60).

### 3.6 Genetic susceptibility

Genetic predisposition significantly influences an individual’s susceptibility to the onset and advancement of oral submucous fibrosis (OSMF) and oral squamous cell carcinoma (OSCC). Genetic variations that affect the metabolism of carcinogens, DNA repair, and the regulation of the cell cycle can lead to different responses to environmental exposures, including areca nut, nicotine, and alcohol (**Figure 2**) (62). Polymorphisms in detoxification enzymes, such as GSTM1 and GSTT1, correlate with diminished efficacy in the elimination of carcinogenic chemicals, resulting in heightened genomic damage inside oral epithelial cells (63). Alterations in tumor suppressor genes such as TP53 impede cellular responses to DNA damage and apoptosis, hence enabling genetically unstable cells to persist (17).

Genetic polymorphisms that impact collagen metabolism and fibrotic pathways may substantially elevate vulnerability to OSMF. Genetic variations associated with extracellular matrix modulation and transforming growth factor signaling may facilitate fibroblast activation and collagen deposition, thus increasing susceptibility to malignant transformation. These data emphasize that genetic predisposition, alongside environmental risk factors, plays a crucial role in the progression of disease from OSMF to OSCC (63, 64).

### 3.7 Epigenetic dysregulation in OSMF-OSCC progression

Epigenetic alterations contribute significantly to the progression of OPMDs and OSCC by regulating gene expression without altering the nucleotide sequence (**Figure 2**). Mechanisms including DNA methylation, histone modifications, and miRNA dysregulation promote genomic instability, altered cell cycle regulation, epithelial proliferation, and malignant transformation (65, 66). Dysregulation of oncogenic and tumor-suppressive miRNAs, including miR-21 and miR-34a, further contributes to tumor progression and therapeutic resistance (67). In addition, epigenetic and miRNA alterations detectable in saliva and blood show promise as non-invasive biomarkers for early disease detection and risk assessment (67, 68). Further mechanistic details regarding epigenetic and transcriptomic plasticity are discussed in Section 5.3.

## 4 Differences in risk exposure and biology between OPMDs and OSCC

The four OPMDs, which include leukoplakia and erythroplakia and oral lichen planus and OSMF, exist as biologically distinct entities that share common risk factors with OSCC yet show different molecular characteristics and degrees of disease advancement. The initial stages of cancer development in OPMDs show oxidative DNA damage and focal p53 accumulation and epigenetic silencing of tumor suppressor genes, but these conditions do not show the chromosomal instability and oncogene amplifications which are typical of invasive carcinoma (4).

Importantly, individual OPMDs exhibit distinct biological and molecular characteristics that influence their patterns of malignant progression. Leukoplakia is more frequently associated with epithelial dysplasia, genomic instability, and field cancerization, whereas erythroplakia demonstrates a higher prevalence of severe dysplasia and early malignant transformation. Oral lichen planus is primarily characterized by chronic immune-mediated inflammation and oxidative stress–associated epithelial injury, while OSMF represents a fibrosis-driven disease model marked by excessive extracellular matrix deposition, tissue stiffness, hypoxia, and mechanotransduction signaling. These lesion-specific differences indicate that malignant transformation in OPMDs occurs through both shared and distinct molecular mechanisms, highlighting the need for individualized risk assessment and targeted therapeutic strategies.

The molecular distinction between two entities establishes OPMDs as temporary evolutionary states which have not yet reached the point of becoming cancerous. The particular pattern of OPMDs which progress to OSCC demonstrates that molecular risk assessment holds critical importance. High-risk OPMDs can be identified by biomarkers such as loss of heterozygosity at specific chromosomal loci, p16 promoter methylation, and elevated miR-21 expression. These markers provide prognostic value that transcends histopathological evaluation, facilitating the initiation of early treatment by clinicians (69).

The OSCC disease progression arises from external carcinogenic influences and internal molecular vulnerabilities, transitioning from healthy mucosal tissue to advanced cancer. Successful prevention measures and early detection methods, as well as targeted treatment methods, require the analysis of risk factors through their biological mechanisms and their impact on human populations (70).

## 5 Molecular mechanisms driving OPMD to OSCC transformation

### 5.1 OPMDs as dynamic pre-cancer states

Oral potentially malignant disorders (OPMDs) consist of a collection of physiologically varied and clinically unique syndromes that may advance to oral squamous cell carcinoma (OSCC). Their growth is dynamic, driven by genetic variations, epigenetic modifications, chronic inflammation, stromal interactions, and environmental exposures, rather than adhering to a fixed sequence (37).

Oral leukoplakia, the predominant oral potentially malignant disorder, exhibits significant heterogeneity. Non-homogeneous lesions, particularly those situated on the tongue or floor of the mouth, possess an elevated risk of malignant development and frequently have increased epithelial dysplasia (71).

Oral submucous fibrosis (OSMF) constitutes a specific condition associated with areca nut consumption. It is characterized by fibrosis, epithelial atrophy, and increased tissue stiffness, which induces cellular stress, genetic instability, and a pro-tumorigenic environment. In OSMF, the risk of malignancy may not have a strong correlation with histological abnormalities (72).

Oral lichen planus (OLP) is an immune-mediated disorder characterized by a diminished overall risk of transformation; however, erosive and atrophic variants demonstrate increased susceptibility. Chronic inflammation, oxidative stress, and recurrent epithelial injury lead to genomic instability and clonal proliferation (72, 73).

Notwithstanding their disparities, OPMDs exhibit shared characteristics, such as modified proliferation, compromised differentiation, persistent inflammation, and premature genetic instability. Field cancerization further facilitates the dissemination of genetically modified cells beyond observable lesions (69, 72). These results emphasize that OPMDs are dynamic conditions requiring continuous monitoring, molecular risk assessment, and immediate intervention to prevent malignant transformation.

### 5.2 Genomic reprogramming and field cancerization

**Figure 3:**
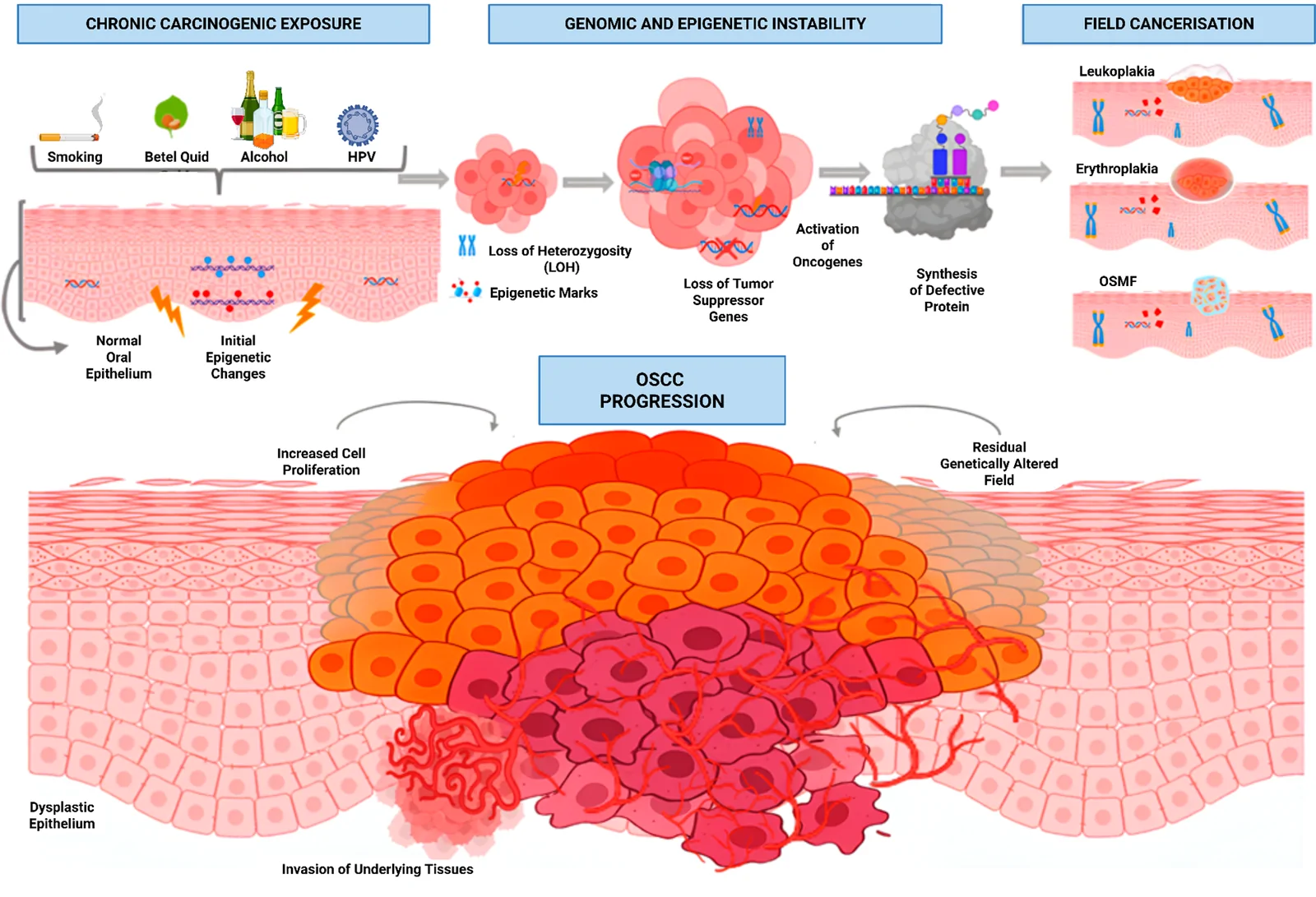
The figure demonstrates the multistep progression of oral carcinogenesis beginning with chronic carcinogen exposure and genomic instability, followed by clonal expansion, epigenetic reprogramming, field cancerization, and eventual progression to invasive OSCC.

Oral potentially malignant diseases (OPMDs) evolve into oral squamous cell carcinoma (OSCC) via an ongoing process of cumulative genomic and epigenetic modifications that initiate prior to the manifestation of histological abnormalities (**Figure 3**). Chronic exposure to carcinogens, including smoke, areca nut, alcohol, and ongoing inflammation, induces the accumulation of genetic mutations and epigenetic alterations, resulting in the disturbance of cellular homeostasis and the proliferation of various genetically modified epithelial clones (69).

These changes mostly influence tumor suppressor pathways that control the cell cycle, respond to DNA damage, and keep the genome stable at the molecular level. Changes in the G1–S checkpoint enable epithelial cells to persist in division despite DNA damage, whereas deficiencies in DNA repair processes exacerbate genomic instability, chromosomal abnormalities, and structural changes that collectively facilitate malignant transformation (69, 74).

Loss of heterozygosity (LOH), which shows the growth of genetically changed clones, is one of the most consistent molecular signs of OPMD progression (**Figure 3**). Significantly, these genetic abnormalities are not limited to clinically apparent lesions but are also seen in neighboring histologically normal mucosa (75). This observation underpins the concept of field cancerization, wherein extensive regions of epithelium experience genetic reprogramming due to extended exposure to carcinogens, leading to the emergence of multiple independent or clonally related lesions and heightening the risk of recurrence and the development of second primary tumors, even following surgical excision (76).

As genetic alterations accumulate, cells exhibit increased aggressiveness, characterized by heightened division, survival signaling, invasion of adjacent cells, and evasion of senescence. The transition from dysplasia to carcinoma is a prolonged, multistage process shaped by both novel and pre-existing genetic alterations, highlighting the gradual nature of oral carcinogenesis (76).

Interestingly, diverse OPMDs such as leukoplakia, oral submucous fibrosis, and oral lichen planus all have different causes and histological characteristics, yet they also show molecular changes that influence cell cycle control, stress response, and chromatin organization. This signifies that diverse initial factors ultimately converge on the identical oncogenic transformation pathway.

Along with genetic changes, epigenetic processes like DNA methylation, histone alterations, and chromatin remodeling are also crucial for accelerating the growth of cancerous cells. These modifications control gene expression without changing the DNA sequence. This process turns off tumor suppressor genes and turns on oncogenes, which keeps the malignant phenotype stable (77).

Generally, the integration of genetic reprogramming with field cancerization alters our perception of oral carcinogenesis from a localized lesion-centric occurrence to a dynamic spatiotemporal phenomenon (78). This paradigm underscores the limitations of conventional diagnostic approaches and emphasizes the imperative for molecular surveillance, targeted therapies, and proactive preventive strategies to effectively manage and reduce the risk of OSCC development.

### 5.3 Epigenetic and transcriptomic plasticity

The advancement of oral potentially malignant disorders (OPMDs) to oral squamous cell carcinoma (OSCC) is influenced by enduring genetic modifications as well as substantial epigenetic and transcriptome alterations (**Figure 3**) (79). Epigenetic pathways facilitate the dynamic regulation of gene expression in response to environmental stimuli, including inflammation and carcinogen exposure. This plasticity enables premalignant epithelial cells to adjust and endure under stress, promoting early transformation while preserving the capacity for malignant progression until stable genetic alterations arise (80).

DNA methylation is a key part of this process, and it has two different patterns as OPMD progresses. Promoter hypermethylation silences tumor suppressor genes that control the cell cycle, apoptosis, and DNA repair (79). This process increases the potential of cancer occurrence. Conversely, global hypomethylation destabilizes the genome by activating proto-oncogenes and repetitive genomic regions, hence altering chromosomes. This imbalance makes the epigenetic environment more open, which speeds up the progression of disease (80, 81).

Histone alterations also control transcriptional activity by changing the shape and accessibility of chromatin. Histone acetylation and methylation changes decide if chromatin stays active or inactive (66, 80, 81). In OPMDs, the dysregulation of histone-modifying enzymes results in the abnormal expression of genes linked to proliferation, survival, and inflammation. These chromatin alterations manifest early, preceding histological dysplasia, underscoring epigenetic reprogramming as a pivotal process in oral carcinogenesis.

Non-coding RNAs, including microRNAs (miRNAs) and long non-coding RNAs (lncRNAs), are important for controlling epigenetic plasticity. miRNAs regulate gene expression post-transcriptionally by targeting various mRNAs, consequently affecting pathways associated with cell proliferation, inflammation, epithelial–mesenchymal transition (EMT), and stem cell properties (82). When miRNAs malfunction, tumor cells exhibit increased longevity and invasiveness. Similarly, lncRNAs regulate chromatin structure, transcription factor activity, and RNA stability, hence perpetuating oncogenic signaling and inhibiting apoptosis.

The combined effects of changes in epigenetics and transcriptomics help premalignant cells gain stem-like traits. These cells acquire the capacity for self-renewal, therapeutic resistance, and adaptation to adverse microenvironments, including hypoxia and immune surveillance. Because epigenetic modifications can be reversed, cells can go from being stem-like to differentiated, which makes phenotypic variability greater and makes it harder to predict how diseases will progress (82).

Significantly, early epigenetic alterations are visible in tissues that appear microscopically normal in regions predisposed to cancer development. This renders them suitable candidates for biomarkers for early diagnosis, risk evaluation, and illness surveillance. Their reversible characteristics render them highly beneficial for chemopreventive therapy aimed at reinstating normal gene expression and halting the advancement of OSCC. In general, OPMDs are dynamic pre-cancerous states caused by interrelated epigenetic and transcriptome pathways that are crucial for malignant transformation (83).

### 5.4 Chronic inflammation as a central driver

The chronic inflammation condition functions as a common biological process that connects environmental factors with the molecular harm seen in oral potentially malignant disorders (OPMDs). Chronic inflammation establishes an ongoing healthcare problem because it creates disordered systems which lead to cancer development, while acute inflammation protects the body through its temporary nature (69). People who use tobacco and areca nut and alcohol and experience microbial attacks in the oral cavity create inflammatory pathways which destroy their epithelial tissues and their ability to maintain stable genes, thus making inflammation the main factor that drives their cancer development.

Long-term exposure to carcinogens in OPMDs causes oxidative and nitrosative stress to last by making reactive oxygen and nitrogen species. These reactive chemicals harm DNA, proteins, and lipids, which causes mutations, mistakes in replication, and problems with repair systems. The invasion of inflammatory cells increases oxidative stress even further, causing a loop of cellular and molecular damage that leads to genetic and epigenetic changes that cause early cancer development (69). Long-term inflammation also turns on important transcription factors that control how cells live and grow. This leads to the constant release of pro-inflammatory cytokines, chemokines, and growth factors, which create autocrine and paracrine signaling between epithelial and stromal cells. As a result, injured cells avoid apoptosis and keep dividing, which creates an imbalance that favors cell growth over cell death. This facilitates the accumulation of oncogenic mutations and the dissemination of cancer.

Chronic inflammatory signaling changes both epithelial cells and the complete immune system present in that area. The immune system becomes exhausted after continuous contact with inflammatory substances, which also transforms how antigens are presented and creates new immunosuppressive cell groups in the oral mucosa (69). These changes decrease immune surveillance functions that normally protect against the emergence of genetically mutated cells. The process of immune tolerance development over time creates an environment which permits genetically modified epithelial cells to expand their population and thrive throughout the tissue area.

The relationship between inflammation and cancer development through multiple pathways leads to increased tumor growth. Inflammatory signaling interacts with epigenetic changes to create abnormal gene expression patterns which maintain cancerous cell characteristics. The system works with genomic instability because it disrupts the DNA repair mechanisms while it causes genetic material to become damaged. Chronic inflammation creates a dual role in oral cancer development by starting the process and maintaining its presence through interconnected biological systems (69, 84).

The findings demonstrate that chronic inflammation drives the development of OPMD because it acts as the primary force behind this process instead of being an effect of tissue damage. The inflammatory microenvironments establish a continuous relationship between environmental exposure and molecular reprogramming, which leads to immune evasion and clonal selection, thus driving OPMD development into cancer. The recognition of inflammation as a pivotal element in this process indicates that anti-inflammatory and immune-modulatory therapies can successfully impede cancer progression from its onset (84).

### 5.5 Tumor microenvironment remodeling

The malignant transition of oral potentially malignant disorders (OPMDs) to oral squamous cell carcinoma (OSCC) is significantly influenced by the dynamic remodeling of the tumor microenvironment (TME), which evolves concurrently with genetically changed epithelial cells. The TME, which includes stromal components, immune cells, and blood vessels, helps tumors grow, invade, and avoid the immune system (85).

Stromal remodeling, in which fibroblasts turn into cancer-associated fibroblasts (CAFs), is an important part. These cells produce excessive extracellular matrix components and release growth factors and cytokines that help epithelial cells grow, survive, and move around. They also cause epithelial–mesenchymal transition (EMT) and make cells more likely to invade (**Table 2**).

**Table 2:** Tumor microenvironment components and their roles in OPMD to OSCC transformation.

Component	Key elements	Functional role in transformation	Clinical relevance
Cancer-associated fibroblasts (CAFs)	Activated fibroblasts, myofibroblasts	ECM remodeling, collagen deposition, EMT induction	Promote fibrosis and tumor progression
Immune cells	T cells, macrophages, neutrophils	Chronic inflammation, immune suppression, cytokine release	Immune evasion, potential immunotherapy targets
Extracellular matrix (ECM)	Collagen, fibronectin, proteoglycans	Tissue stiffness, mechanotransduction (YAP/TAZ activation)	Key driver in OSMF-associated carcinogenesis
Cytokines and growth factors	TGF-β, IL-6, TNF-α	Inflammation, fibroblast activation, signaling crosstalk	Link inflammation to cancer progression
Endothelial cells	VEGF, angiogenic factors	Angiogenesis, tumor vascularization	Supports tumor growth and metastasis
Hypoxia-related factors	HIF-1α	Adaptation to low oxygen, metabolic reprogramming	Promotes aggressive tumor phenotype
Extracellular vesicles (Exosomes)	miRNAs, proteins, lipids	Cell–cell communication, microenvironment modulation	Non-invasive biomarkers, therapeutic targets

ECM remodeling and mechanotransduction signaling, particularly in OSMF, further contribute to malignant progression, as discussed in Section 5.7. The tumor microenvironment further promotes immune evasion and chronic inflammatory signaling, as discussed in Section 5.4 (85).

Angiogenic remodeling results in the development of aberrant blood vessels, inducing hypoxic and nutrient-deficient environments that favor aggressive and adaptable cancer cell morphologies (86). In general, the interaction between stromal, immunological, and vascular components provides a self-sustaining, pro-tumorigenic environment with geographic heterogeneity. This phenomenon makes the TME a key factor in the evolution of OPMD and a possible target for treatment.

The cellular components of the tumor microenvironment interact through complex bidirectional signaling networks that actively drive malignant progression. Cancer-associated fibroblasts (CAFs) secrete cytokines, growth factors, and extracellular matrix components that promote epithelial proliferation, epithelial–mesenchymal transition (EMT), and matrix remodeling. In turn, transformed epithelial cells release inflammatory mediators and extracellular vesicles that further activate fibroblasts and modulate immune cell behavior. Immune cells, including tumor-associated macrophages and T cells, contribute to a chronic inflammatory and immunosuppressive microenvironment through secretion of IL-6, TNF-α, TGF-β, and other cytokines that enhance tumor survival and immune evasion. Simultaneously, endothelial cells respond to hypoxia-induced VEGF signaling to promote angiogenesis, while extracellular matrix stiffening activates mechanotransduction pathways such as YAP/TAZ, reinforcing invasive and pro-tumorigenic cellular behavior. These reciprocal interactions collectively establish a dynamic microenvironment that facilitates OPMD-to-OSCC transformation.

Emerging evidence also highlights the role of immune checkpoint pathways, including PD-1/PD-L1 and CTLA-4 signaling, in facilitating immune evasion during OPMD-to-OSCC progression (87). Increased PD-L1 expression in dysplastic lesions and OSCC has been associated with T-cell exhaustion, suppression of antitumor immunity, and disease progression, suggesting potential relevance for immunotherapeutic strategies (88).

### 5.6 Convergent hallmarks of malignant transformation

**Figure 4:**
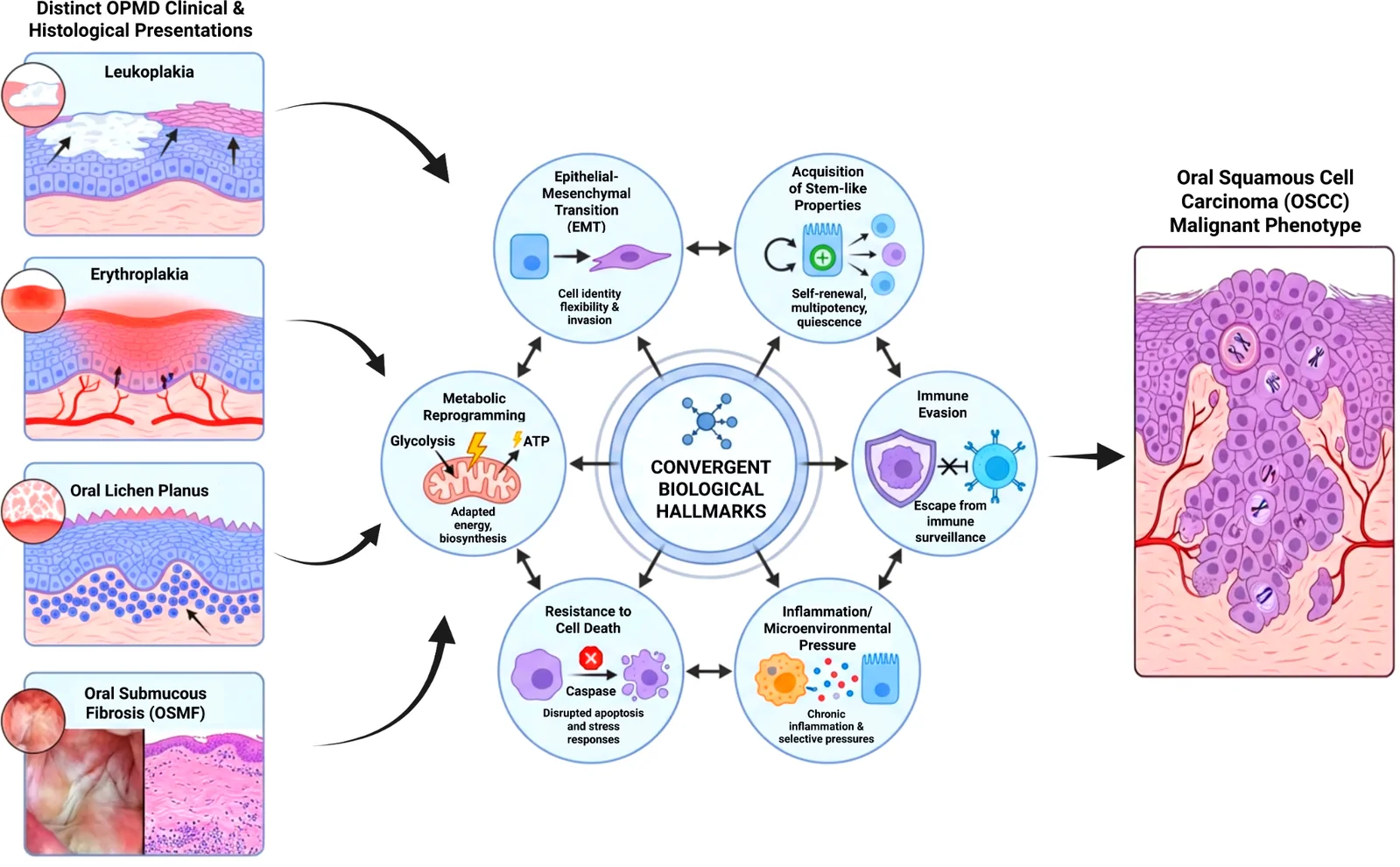
The figure summarizes the major biological hallmarks shared during OPMD to OSCC progression, including epithelial–mesenchymal transition, stem-like properties, metabolic reprogramming, immune evasion, and resistance to apoptosis.

The clinical presentation of oral potentially malignant disorders (OPMDs) shows major differences, which extend to their histopathological features and their underlying causes, but the OPMDs develop towards OSCC through a common set of biological characteristics. The different precursor lesions develop into particular malignant phenotypes because environmental factors and ongoing inflammation and immune system monitoring and tissue-specific conditions create constant pressure on the lesions (37, 86). The common biological characteristics unite various OPMDs through which researchers study their development into advanced disease stages and assess their chances for developing cancer (**Figure 4**).

The first major event that leads to common development processes between different cell types occurs when epithelial-mesenchymal transition (EMT) programs start. The process of epithelial-mesenchymal transition (EMT) allows epithelial cells to change their complete identity into a state where they can move through space and invade other areas while staying alive. OPMDs show a mixed existence of epithelial and mesenchymal traits through which cells can develop their capacity to invade body tissue while retaining their ability to grow through epithelial pathways (37). The flexible endothelial-mesenchymal transition state enables cells to invade nearby tissues while they develop resistance to programmed cell death and gain the ability to endure oxidative stress and mechanical stress, which brings them closer to cancerous development.

The process of acquiring stem-like properties directly continues EMT, which is a common feature characterizing all OPMDs during disease progression. The process of epigenetic reprogramming together with inflammatory signaling pathways initiates the development of self-renewal capacity and multipotent abilities, which provide organisms with extended lifespan advantages (37). The stem-like epithelial cells exhibit enhanced resistance against DNA damage, immunological assaults, and medicinal interventions. The capacity of these cells to remain dormant or progress slowly allows them to persist in regions exhibiting initial signs of cancer development, thereby accumulating further oncogenic genetic alterations over time. The presence of these specific cells in OPMDs raises the risk of cancer development and treatment-related recurrence.

The process of metabolic reprogramming serves as a shared pathway that links all aspects of cancer development. Epithelial cells develop new metabolic pathways when they encounter hypoxic conditions and face variations in nutrient availability and experience greater demands for cell growth. The modified energy metabolism system enables cells to grow quickly while maintaining redox balance and producing essential biosynthetic materials necessary for survival in extremely challenging microenvironmental conditions. The metabolic changes enable cancer cells to grow but they also affect their epigenetic processes and their relationship with the immune system which makes cancerous traits stronger in multiple OPMD types.

As OPMDs progress, a shared survival strategy of immune evasion becomes evident. Persistent inflammatory signaling contributes to immune dysfunction and malignant progression (86). Epithelial cells undergo alterations that reduce their immunogenicity while exploiting the immunosuppressive microenvironment. This allows them to evade cytotoxic lymphocyte–mediated destruction and expand through clonal proliferation. Consequently, the immune system loses its ability to eliminate genetically and epigenetically altered cells, enabling their persistence and further malignant transformation.

Resistance to cell death is another defining feature of OPMDs. Damaged epithelial cells sustain survival by disrupting apoptotic pathways and stress-response mechanisms, allowing them to persist despite selective pressures. This promotes cancer development, as these cells acquire resistance to programmed cell death and, upon transformation, may also exhibit therapy resistance (86). These processes are highly interconnected: epithelial–mesenchymal transition enhances stem-like properties and immune evasion, metabolic reprogramming supports survival under stress, and immune dysfunction further facilitates tumor progression. Such interconnected adaptations drive a convergent molecular pathway, explaining how diverse precursor lesions ultimately progress to oral squamous cell carcinoma with similar phenotypic features.

The identification of shared malignant transformation traits brings important effects to medical practice. The assessment of risk now depends on evaluating pathways instead of evaluating specific lesions, which improves our ability to forecast cancer development (89). The approach to disease prevention and treatment development should focus on common disease characteristics instead of particular disease causes. The combined malignant characteristics of oral carcinogenesis demonstrate that this process follows evolutionary development, which results from natural selection instead of random occurrences. The development of oral cancer results from common disease pathways which all OPMDs follow despite their different origins (**Table 3**) (89).

**Table 3:** Key signaling pathways driving malignant transformation from OPMDs to OSCC.

Pathway	Key components	Functional role	Clinical relevance
TGF-β/SMAD	TGF-β1, SMAD2/3/4	Fibrosis, EMT, ECM deposition	Central driver in OSMF; anti-fibrotic target
PI3K/AKT/mTOR	PI3K, AKT, mTOR	Cell survival, proliferation, metabolism	Major oncogenic pathway; therapeutic target
MAPK	ERK, JNK, p38	Proliferation, stress response	Associated with tumor growth and progression
NF-κB	TNF-α, IL-6	Chronic inflammation, survival signaling	Links inflammation to carcinogenesis
Wnt/β-catenin	β-catenin, APC	Stemness, proliferation, EMT	Tumor initiation and progression marker
YAP/TAZ (Hippo)	YAP, TAZ, TEAD	Mechanotransduction, ECM stiffness	Key in fibrosis-driven carcinogenesis
Hypoxia (HIF-1α)	HIF-1α, VEGF	Angiogenesis, metabolic adaptation	Promotes tumor survival and invasion
Hedgehog	SHH, SMO, GLI	Stemness, proliferation	Emerging pathway in tumor progression
Apoptosis/Cell Cycle	p53, Bcl-2, Cyclin D1	Cell survival, proliferation control	Dysregulation leads to malignant transformation
DNA Damage Response	ATM, ATR, BRCA	Genomic stability, DNA repair	Mutation accumulation and cancer progression
EMT Regulators	Snail, Twist, E-cadherin	Invasion, metastasis	Marker of aggressive phenotype

### 5.7 Mechanotransduction and ECM stiffness (YAP/TAZ signaling)

Mechanotransduction, the mechanism via which cells detect and react to mechanical stimuli, is pivotal in the advancement of oral submucous fibrosis (OSMF) to oral squamous cell carcinoma (OSCC). Excessive collagen accumulation and crosslinking in OSMF render the extracellular matrix (ECM) more rigid, activating mechanosensitive pathways such as YAP/TAZ. Integrins and focal adhesions send mechanical signals that create tension in the cytoskeleton and block the Hippo route (90). This lets YAP/TAZ go into the nucleus. YAP/TAZ interact with TEAD transcription factors in the nucleus to control genes that are important for cell growth, survival, and remodeling of the matrix (**Table 3**).

In OSMF, continuous activation of YAP/TAZ stimulates fibroblast proliferation, myofibroblast differentiation, and heightened ECM synthesis, establishing a positive feedback loop that further increases tissue stiffness. In epithelial cells, YAP/TAZ signaling facilitates cell proliferation, inhibits apoptosis, and encourages epithelial–mesenchymal transition (EMT), hence contributing to malignant transformation. It also works with other signaling pathways, such as TGF-β, Wnt/β-catenin, and PI3K/AKT, to make signals that promote tumors stronger. During the transition from OSMF to OSCC, persistent ECM stiffening and nuclear localization of YAP/TAZ contribute to tumor growth, invasion, and angiogenesis, highlighting this pathway as a potential therapeutic target (31).

## 6 Signaling pathways in OSMF to OSCC transformation

**Figure 5:**
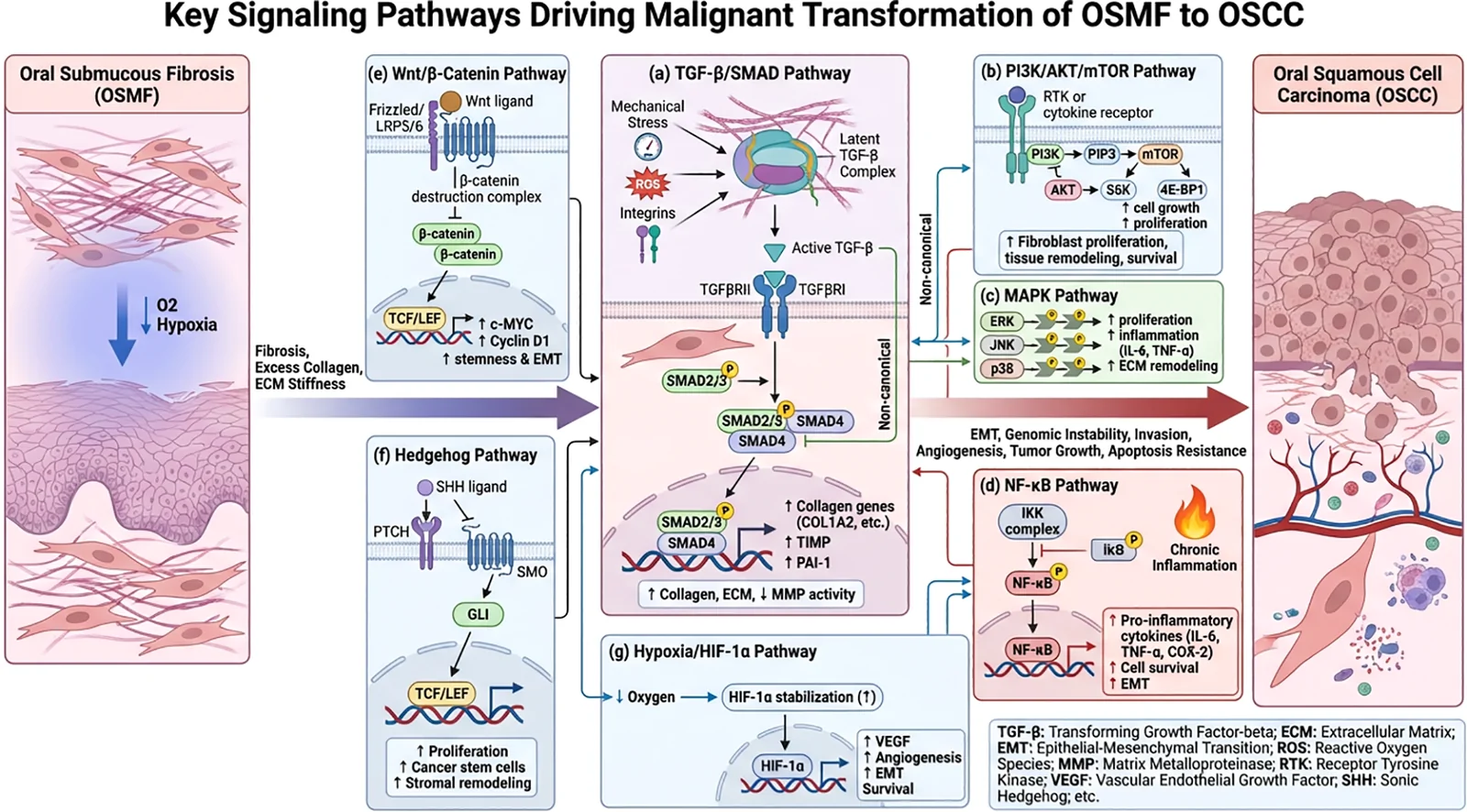
The figure illustrates the interconnected signaling pathways involved in OSMF to OSCC progression. Chronic areca nut exposure and oxidative stress initially activate TGF-β/SMAD signaling, leading to fibrosis and extracellular matrix remodeling. Persistent inflammation and hypoxia subsequently activate PI3K/AKT, MAPK, NF-κB, Wnt/β-catenin, and YAP/TAZ signaling pathways that collectively promote epithelial mesenchymal transition, immune evasion, proliferation, invasion, and malignant transformation.

Malignant transformation of oral submucous fibrosis (OSMF) into oral squamous cell carcinoma (OSCC) is driven by the interplay of multiple signaling pathways that regulate fibrosis, inflammation, cellular proliferation, and epithelial–mesenchymal transition (EMT) (91). Chronic exposure to areca nut alkaloids, oxidative stress, and persistent inflammation leads to sustained activation of profibrotic and oncogenic signaling networks. Among these, the TGF-β/SMAD pathway represents the central regulator of fibrosis, while additional pathways including PI3K/AKT, MAPK, NF-κB, Wnt/β-catenin, Hedgehog, and hypoxia-induced signaling cooperate to promote malignant transformation (**Figure 5**) (92).

These signaling pathways do not function independently but instead exhibit extensive crosstalk and stage-specific activation during OSMF-to-OSCC progression. TGF-β/SMAD signaling acts as a major upstream profibrotic regulator during the early stages of fibrosis by promoting extracellular matrix deposition and fibroblast activation. Persistent fibrotic stress, chronic inflammation, and oxidative damage subsequently activate downstream oncogenic pathways, including PI3K/AKT, MAPK, NF-κB, and Wnt/β-catenin signaling, which collectively enhance epithelial–mesenchymal transition, proliferation, immune evasion, and malignant transformation. Importantly, these pathways converge at multiple molecular nodes to sustain a pro-tumorigenic microenvironment and reinforce malignant progression. In advanced stages, mechanotransduction pathways such as YAP/TAZ further interact with these signaling networks in response to extracellular matrix stiffening, thereby sustaining tumor progression and invasive behavior.

### 6.1 TGF-β/SMAD signaling pathway in OSMF

Transforming growth factor-β (TGF-β) is a multifunctional cytokine that regulates cellular proliferation, differentiation, immune modulation, and extracellular matrix (ECM) homeostasis. Among the cytokines involved in fibrotic disorders, TGF-β acts as the master regulator of fibrogenesis (**Figure 5**). Persistent activation of TGF-β signaling in oral tissues results in excessive collagen deposition, suppression of ECM degradation, and progressive tissue rigidity, which are hallmark features of oral submucous fibrosis (OSMF) (92).

Three isoforms of TGF-β have been identified in mammals TGF-β1, TGF-β2, and TGF-β3, encoded by the genes *TGFB1*, *TGFB2*, and *TGFB3*. Among these, TGF-β1 is the predominant isoform associated with fibrosis and is significantly elevated in OSMF tissues (**Table 3**) (93).

#### 6.1.1 Activation of TGF-β signaling

TGF-β is secreted as a latent complex bound to the latency-associated peptide (LAP) and stored within the extracellular matrix. Activation of latent TGF-β occurs through mechanical stress, reactive oxygen species, proteolytic enzymes, and integrin-mediated interactions (particularly αvβ6 and αvβ8 integrins). In OSMF, chronic areca nut exposure and oxidative stress promote activation of this latent cytokine (93).

Following activation, TGF-β binds to type II receptors (TGF-βRII) on the cell surface, which subsequently recruit and phosphorylate type I receptors (TGF-βRI). This receptor complex initiates intracellular signaling cascades (94). The canonical signaling pathway involves phosphorylation of SMAD2 and SMAD3, which then associate with SMAD4 to form transcriptional complexes that translocate into the nucleus. These complexes regulate genes involved in extracellular matrix production, fibroblast activation, and immune modulation. In addition to SMAD signaling, TGF-β also activates non-canonical pathways, including MAPK, PI3K/AKT, and Rho-GTPase signaling, which enhance fibroblast proliferation, cytoskeletal remodeling, and extracellular matrix synthesis (18, 94).

#### 6.1.2 Collagen production and ECM synthesis

One of the primary indications for OSMF occurs when excessive amounts of extracellular matrix, especially collagen, build up. TGF-β signaling activates the transcription of several collagen genes, including COL1A2, COL3A1, COL6A1, COL6A3, and COL7A1, which results in the production of more fibrillar collagens. Collagen types I and III make up most of the fibrotic extracellular matrix. They make thick bundles of collagen that make tissues less elastic.

Procollagen molecules that have just been made go through post-translational processing in the extracellular matrix. TGF-β increases the activity of procollagen processing enzymes, such as procollagen N-proteinase (PNP) and procollagen C-proteinase (PCP/BMP1). These enzymes take propeptides out of procollagen to make mature tropocollagen that can form fibrils.

Cross-linking mediated by lysyl oxidase (LOX) makes collagen fibers even stronger. LOX is an enzyme that needs copper to work. It catalyzes the oxidative deamination of lysine residues, which makes covalent cross-links that make collagen fibers stronger. Chewing areca nuts raises the amount of copper in the tissues of the mouth, which in turn boosts LOX activity and helps build collagen networks that are difficult to break down (95).

As cross-linked collagen builds up, it makes the mucosa stiffer, the epithelium shrinks, and the blood vessels become less dense. This creates a low-oxygen environment that encourages fibrotic signaling even more.

#### 6.1.3 Suppression of collagen degradation (TIMPs and PAI-1)

Matrix metalloproteinases (MMPs) are vital to preserving the stability of the extracellular matrix (ECM) as they degrade collagen and other matrix components. In oral submucous fibrosis (OSMF), transforming growth factor-β (TGF-β) disrupts this balance by inhibiting collagen breakdown (96). This occurs through two primary molecular mechanisms. Initially, elevating the levels of tissue inhibitors of metalloproteinases (TIMPs) directly inhibits active MMPs, hence decelerating the destruction of the extracellular matrix (ECM). Second, inducing plasminogen activator inhibitor-1 (PAI-1) stops plasminogen from turning into plasmin, which is a necessary step for turning on latent MMPs. Overexpressing both TIMPs and PAI-1 creates a dual inhibitory mechanism that stops ECM degradation, which leads to more collagen building up and fibrosis (96).

### 6.2 PI3K/AKT/mTOR pathway

The phosphatidylinositol-3-kinase (PI3K)/AKT/mammalian target of rapamycin (mTOR) signaling pathway is a key controller of cell growth, survival, metabolism, and angiogenesis. Its malfunction is a major factor in the progression from oral submucous fibrosis (OSMF) to oral squamous cell carcinoma (OSCC) (96, 97). In OSMF, prolonged exposure to areca nut constituents, oxidative stress, and inflammatory cytokines results in the sustained activation of PI3K signaling (**Figure 5**). When receptor tyrosine kinases are activated, they cause PI3K to convert PIP2 into PIP3, which then brings in and activates AKT. When AKT is activated, it adds a phosphate group to many downstream targets, including mTOR, which is a crucial effector that boosts protein synthesis through S6K and 4E-BP1.

This promotes cellular growth and reduces their susceptibility to apoptosis. This pathway in fibroblasts stimulates proliferation and excessive extracellular matrix deposition, resulting in fibrosis. In epithelial cells, it facilitates the survival of genetically modified cells and the accumulation of oncogenic mutations. Additionally, PI3K/AKT signaling interacts This pathway interacts with other pathways, including TGF-β, MAPK, and hypoxia-inducible pathways, to facilitate epithelial–mesenchymal transition, angiogenesis, and metabolic adaptability (**Table 3**). Hyperactivation of this pathway is a hallmark of malignant transformation, as seen by elevated phosphorylated AKT and mTOR levels in OSCC tissues. The PI3K/AKT/mTOR axis is a critical therapeutic target because inhibitors of this system may prevent high-risk OSMF lesions from progressing to cancer (96, 97).

### 6.3 MAPK signaling

The mitogen-activated protein kinase (MAPK) signaling pathway is crucial for changing how cells respond to stresses in the environment, growth hormones, and cytokines. The MAPK pathway has three main kinase cascades: the extracellular signal-regulated kinase (ERK), the c-Jun N-terminal kinase (JNK), and the p38 MAPK pathways (**Figure 5**). These signaling modules regulate cellular growth, differentiation, apoptosis, and inflammatory responses (98).

In oral submucous fibrosis (OSMF), prolonged exposure to areca nut alkaloids and reactive oxygen species promotes MAPK signaling in oral epithelial cells and fibroblasts. The stimulation of the ERK pathway promotes cellular proliferation and enhances fibroblast activity, hence enabling extracellular matrix production and tissue remodeling (98). The JNK and p38 MAPK pathways are mostly involved in signaling for inflammation and stress responses. This results in the increased production of pro-inflammatory cytokines and growth factors.

Continual activation of MAPK signaling can promote epithelial cell proliferation and survival, hence aiding the proliferation of genetically modified cells within a fibrotic environment. Additionally, MAPK signaling interacts with TGF-β signaling pathways to enhance fibroblast activation and collagen production (**Table 3**) (98). This interaction helps maintain the structure of fibrotic tissue and promotes epithelial–mesenchymal transition (EMT). During the progression from oral submucous fibrosis (OSMF) to oral squamous cell carcinoma (OSCC), dysregulated MAPK signaling promotes unchecked cellular proliferation, resistance to apoptosis, and increased invasive potential. Overactivation of ERK signaling has been observed in OSCC tissues and is associated with enhanced tumor development and metastasis (79).

### 6.4 NF-κB inflammatory pathway

Nuclear factor-κB (NF-κB) is a key transcription factor that controls inflammation, immune responses, and cell viability. Inflammatory cytokines, oxidative stress, microbial products, and other environmental stressors usually turn on the NF-κB signaling pathway (99). In OSMF, chronic inflammation caused by areca nut consumption and oxidative stress leads to persistent activation of NF-κB signaling in oral mucosal tissues (**Figure 5**). Activation of this system results in increased production of pro-inflammatory cytokines, including tumor necrosis factor-α (TNF-α), interleukin-6 (IL-6), and cyclooxygenase-2 (COX-2). These inflammatory mediators help fibroblasts become active, bring immune cells to the site of inflammation, and keep the inflammation going in the oral mucosa (**Table 3**).

The NF-κB signaling system makes epithelial cells live longer by making proteins that stop cells from dying and encouraging cells to divide. This system is constantly active, resulting in an inflammatory environment that facilitates tumor development and induces genomic instability and malignancy (99). NF-κB also interacts with other signaling pathways that are critical for fibrosis and cancer, such as the TGF-β, PI3K/AKT, and MAPK pathways. This signaling network helps OSCC grow and spread by assisting with epithelial-mesenchymal transition, angiogenesis, and immune evasion.

### 6.5 Wnt/β-catenin pathway

The Wnt/β-catenin signaling system is essential for determining how cells change, how stem cells stay alive, and how tissues are made. The canonical Wnt signaling pathway stops the breakdown of β-catenin by having Wnt ligands and Frizzled receptors work together with LRP5/6 co-receptors (**Figure 5**). Consequently, β-catenin accumulates in the cytoplasm and translocates to the nucleus, where it initiates the transcription of genes critical for cellular development and survival (99).

In OSMF, alterations in Wnt signaling can make epithelial cells behave strangely and generate more of them. When β-catenin-mediated transcription is turned on, it makes genes that cause cancer, such as c-MYC and cyclin D1, work harder. These genes help cells move through the cell cycle and build tumors (**Table 3**) (92, 99). In addition, Wnt/β-catenin signaling is critical for keeping cancer stem cells alive and for the process of epithelial–mesenchymal transition. These processes are especially vital for the initiation, invasion, and dissemination of malignancies. The irregular activation of Wnt signaling throughout the progression from OSMF to OSCC can facilitate malignant transformation and tumor growth.

### 6.6 Hedgehog signaling

The Hedgehog signaling mechanism is crucial for the growth of embryos and the repair of tissues. When hedgehog ligands, such as Sonic hedgehog (SHH), bind to the Patched (PTCH) receptor, they start this process by stopping the inhibition of the transmembrane protein Smoothened (SMO). When SMO is turned on, GLI transcription factors move into the nucleus, where they control the production of genes that are important for cell growth and differentiation (**Table 3**) (100).

In oral submucous fibrosis (OSMF), dysregulated Hedgehog signaling activity correlates with heightened epithelial proliferation and stromal remodeling. This route may lead to increased fibroblast activity and changes in the structure of the tissue in the fibrotic mucosa. Moreover, Hedgehog signaling plays a role in preserving cancer stem cell populations and facilitating tumor growth during malignant transformation (**Figure 5**). Oral malignancies have been found to have too many Hedgehog pathway components, which may play a role in starting and growing tumors (100, 101).

### 6.7 Hypoxia and HIF-1α signaling

Progressive fibrosis in oral submucous fibrosis (OSMF) results in diminished vascularization and compromised oxygen transport inside the mouth mucosa. This creates a low-oxygen environment that turns on hypoxia-inducible factor-1α (HIF-1α), which is an important transcription factor that controls how cells adapt to low oxygen levels (100–102).

The activation of HIF-1α enhances the transcription of genes associated with angiogenesis, metabolic adaptability, and cellular survival, such as vascular endothelial growth factor (VEGF). Hypoxia-induced signaling promotes epithelial–mesenchymal transition and augments the invasive potential of epithelial cells. The hypoxic milieu created by extensive collagen accumulation thereby facilitates the malignant evolution of OSMF (**Figure 5**). The interaction between HIF-1α signaling and pathways like TGF-β, PI3K/AKT, and NF-κB enhances tumor-promoting signals and promotes the advancement to OSCC (102). The major signaling pathways involved in the malignant transformation of OPMDs to OSCC are summarized in **Table 3**.

## 7 Biomarkers and precision risk stratification

The major challenge in oral oncology lies in predicting which patients with oral potentially malignant disorders (OPMDs) will progress to malignancy. Conventional clinical and histopathological assessments fail to capture the full biological complexity and progression patterns of these lesions, emphasizing the necessity of reliable risk assessment biomarkers. Tissue-based biomarkers remain central to molecular evaluation, as genetic alterations affecting cell cycle regulation, apoptosis, DNA repair, and differentiation drive malignant transformation (103). Molecular profiling of biopsy samples enables early detection of genomic and epigenetic changes before visible transformation and also provides spatial information about high-risk cellular regions. However, sampling bias and the requirement for repeated invasive procedures limit these approaches.

Circulating biomarkers, including cell-free DNA and RNA, have emerged as a promising alternative, offering a non-invasive means to detect molecular alterations across both visible and occult lesions. These biomarkers reflect genetic, epigenetic, and transcriptional changes occurring throughout the malignant field, even at early stages (104). Multi-omics data encompassing genomic, epigenomic, transcriptomic, proteomic, and metabolomic layers integrate to provide a comprehensive understanding of OPMD biology. When combined with machine learning approaches, such data enable the development of predictive models capable of distinguishing lesions with high malignant potential from those likely to remain stable. Beyond prediction, biomarker-based risk stratification supports personalized clinical management, allowing early intervention for high-risk lesions while avoiding unnecessary treatment in low-risk cases (105).

Genetic biomarkers, including somatic mutations, loss of heterozygosity, and copy number variations, serve as early indicators of irreversible genomic damage. Epigenetic alterations, such as aberrant DNA methylation and histone modifications, often precede visible dysplasia and contribute to transcriptional dysregulation and genomic instability (105). Transcriptomic biomarkers, including mRNAs and non-coding RNAs, reflect dynamic cellular states and regulate pathways associated with proliferation, inflammation, epithelial-mesenchymal transition, and stemness. Proteomic biomarkers further reveal active cellular processes, including alterations in cell cycle regulation, angiogenesis, inflammation, and extracellular matrix remodeling (**Table 4**). Collectively, the integration of these biomarker types enables a robust and dynamic framework for risk stratification, shifting OPMD management from conventional static assessment toward precision-based, personalized early intervention strategies (106).

**Table 4:** Biomarkers for early detection and risk stratification in OPMDs and OSCC.

Biomarker type	Examples	Source	Clinical utility
Genetic	TP53 mutations, LOH	Tissue, ctDNA	Early detection, mutation profiling
Epigenetic	p16 methylation, MGMT	Tissue, saliva	Risk prediction, gene silencing
miRNA	miR-21, miR-155, miR-31	Saliva, blood, exosomes	Non-invasive diagnosis
Proteomic	EGFR, Cyclin D1, p53	Tissue, exosomes	Prognosis, tumor progression
Metabolomic	Lactate, amino acids	Serum, saliva	Functional metabolic changes
Exosomal	miRNAs, proteins	Blood, saliva	Real-time monitoring, early detection
Circulating biomarkers	cfDNA, ctDNA	Blood	Disease progression tracking

### 7.1 Tissue biomarkers

Tissue-based biomarkers remain the foundation of molecular risk assessment in OPMDs, as they enable direct evaluation of lesion-specific alterations within the epithelial and stromal compartments (**Table 4**) (107). These biomarkers give important geographical information that makes it possible to find high-risk cellular subclones in lesions that are histologically diverse. Importantly, tissue profiling can uncover early molecular alterations that are undetected by standard histological evaluation. However, their clinical applicability is constrained by sample bias and the necessity for repeated invasive operations, especially in lesions with field cancerization (107).

### 7.2 Salivary biomarkers

Tissue-based biomarkers are still crucial for the molecular diagnosis of oral potentially malignant disorders (OPMDs) because they give important spatial information and help find high-risk cellular subclones in lesions that are histologically heterogeneous (108). Proliferation markers like Ki-67, tumor suppressors like p53, cell cycle regulators like cyclin D1, and indicators of epithelial dysplasia like p16 are some of the most common tissue biomarkers that scientists look at. Changes in signaling molecules including β-catenin and EGFR have also been linked to a higher likelihood of malignant transformation (**Table 4**). Tissue profiling can reveal early molecular changes that are not evident through routine histopathological assessment. However, their clinical value is constrained by sample bias and the necessity for repeated invasive operations, especially in lesions demonstrating field cancerization (109).

### 7.3 Liquid biopsy biomarkers

Liquid biopsy biomarkers, especially circulating cell-free DNA (cfDNA), circulating tumor DNA (ctDNA), and circulating microRNAs, offer a minimally invasive method for evaluating molecular changes linked to oral potentially malignant disorders (OPMDs) (**Table 4**). These biomarkers indicate genetic and epigenetic alterations derived from both clinically evident lesions and adjacent modified mucosa, hence reinforcing the notion of field cancerization (109, 110).

Detecting abnormalities, including TP53 mutations, DNA methylation modifications, and dysregulated circulating microRNAs (e.g., miR-21 and miR-31), has been linked to a higher risk of malignant transformation. Liquid biopsy has the benefit over tissue-based approaches of being able to capture changes that happen over time and in the whole body. This makes it particularly effective for early disease detection, assessing the likelihood of disease progression, and monitoring disease progression (110).

Despite its significant potential, challenges remain in terms of standardization, sensitivity, and validation in large groups. Nonetheless, ongoing advancements in detection technology are anticipated to improve the clinical utility of liquid biopsy in forecasting the transition from oral potentially malignant disorders (OPMDs) to oral squamous cell carcinoma (OSCC) (103).

### 7.4 Exosomal biomarkers

Exosomes are tiny vesicles that epithelial, stromal, and immunological cells release into the extracellular space. They help cells talk to each other in the tumor microenvironment. In oral potentially malignant disorders (OPMDs), exosomal cargo signifies underlying genetic modifications linked to disease advancement (111). These vesicles contain a variety of bioactive substances, such as proteins, messenger RNAs, microRNAs, and lipids, that affect important processes like inflammation, epithelial plasticity, and remodeling of the extracellular matrix (**Table 4**).

Exosomal microRNAs such as miR-21, miR-155, and miR-1246 have been linked to tumor growth, changes in the immune system, and the transition from epithelial to mesenchymal cells. Moreover, exosomal proteins, including epidermal growth factor receptor (EGFR) and heat shock proteins, have been associated with increased cell proliferation and stress response pathways. Exosomes highlight the interaction between epithelial and stromal cells, enhancing our comprehension of the temporal alterations in the lesion’s microenvironment (111).

One of the best circumstances about exosomal biomarkers is that they stay stable in biological fluids like blood and saliva, which makes them suitable for non-invasive detection and long-term monitoring. Their role in enhancing epithelial–stromal contact identifies them as functional biomarkers that not only indicate disease status but may also contribute to malignant transformation. However, prior to its implementation in routine clinical practice, issues pertaining to isolation techniques, standardization, and clinical validation must be resolved (112).

Exosomal biomarkers hold significant potential for early detection and risk evaluation in oral potentially malignant disorders (OPMDs), establishing a functional connection between the microenvironment and the advancement to oral squamous cell carcinoma (OSCC).

### 7.5 Metabolomic biomarkers

Metabolomic biomarkers elucidate the biochemical changes linked to oral potentially malignant disorders (OPMDs) and their advancement to oral squamous cell carcinoma (OSCC). Metabolic reprogramming is a defining characteristic of carcinogenesis, indicating alterations in cellular energy generation, redox equilibrium, and biosynthetic pathways (113). In OPMDs, these changes can be found in biological fluids, including saliva, serum, and tissue samples. This analysis provides a functional assessment of molecular alterations associated with the condition (**Table 4**).

Amino acids, lipids, and intermediates of glycolysis and the tricarboxylic acid (TCA) cycle are only a few of the metabolite types that have been linked to the course of OPMD. Higher amounts of lactate, alterations in amino acid profiles (such as glutamine and serine), and changes in lipid metabolism have all been linked to faster cell growth and the ability to adapt to low oxygen levels. These metabolic changes help cells develop quickly and help create a microenvironment that is good for tumors (114).

Metabolomic profiling makes it possible to identify unique metabolic fingerprints that can tell the difference between high-risk lesions and low-risk diseases. Metabolomic biomarkers improve the accuracy of disease prediction and provide useful information about changes in functional pathways when used with other molecular data. However, variations in sample collection methods, analytical procedures, and data interpretation persist in complicating clinical translation (114). Metabolomic markers are a viable methodology for the early detection of OPMDs and the assessment of their associated risks. They facilitate a deeper comprehension of malignant transformation by improving the integration of genomic and proteomic data.

### 7.6 Multi-omics biomarker integration

Multi-omics biomarker integration offers a holistic framework for elucidating the intricate molecular pathways that drive the advancement of oral potentially malignant disorders (OPMDs) to oral squamous cell carcinoma (OSCC) (114, 115). When looked at alone, each layer of biological data doesn’t provide us much information. However, by integrating genomic, epigenomic, transcriptomic, proteomic, and metabolomic data, we can attain a more comprehensive understanding of disease mechanisms. This method shows the interplay of several regulatory levels, facilitating the identification of primary reasons for malignant transformation.

Integrated analysis enables the precise identification of convergent signaling pathways and molecular networks implicated in proliferation, apoptosis, fibrosis, and immunological regulation. Multi-omics profiling facilitates the identification of robust biomarker signatures that enhance risk stratification and differentiate between high-risk and low-risk lesions (114–116). This systems-level approach is important because it helps find early molecular changes that happen before histopathological changes, which makes early intervention more likely.

Adding computational methods like machine learning and network-based modeling makes multi-omics integration even stronger by letting you look at complicated datasets and making predictions more accurate. This makes it possible to create tailored risk models that take into account clinical, molecular, and environmental aspects. Nevertheless, obstacles such as data harmonization, integration complexity, and validation across heterogeneous populations continue to pose substantial impediments to clinical translation (115, 116). The integration of multi-omics biomarkers is a significant advancement for precision medicine in OPMDs. It provides a systems-level perspective on illness progression and facilitates novel approaches for early detection, risk prediction, and targeted treatments.

Recent advances in single-cell sequencing and spatial transcriptomics have provided deeper insights into the cellular heterogeneity and spatial organization of OPMDs and OSCC. These technologies enable characterization of epithelial, stromal, and immune cell populations at single-cell resolution while preserving tissue architecture, thereby improving understanding of tumor evolution, immune interactions, and microenvironmental remodeling during malignant transformation (117, 118).

Although several biomarkers have shown promising diagnostic and prognostic potential in OPMD and OSCC research, many findings are currently derived from preclinical studies or small patient cohorts, limiting their immediate clinical applicability. Differences in study design, lesion heterogeneity, analytical platforms, and validation methods have contributed to variability and inconsistent reproducibility across studies. Consequently, only a limited number of biomarkers have progressed toward large-scale clinical validation for routine diagnostic or prognostic use, highlighting the translational gap between experimental biomarker discovery and clinical implementation.

## 8 AI-based prediction models

AI-based prediction models are becoming more useful for better risk stratification and finding oral potentially malignant diseases (OPMDs) early. Standard clinical and tissue tests sometimes do not clearly explain the complex biological processes that lead to cancer. Conversely, AI methodologies facilitate the analysis and manipulation of extensive, multi-dimensional datasets (119). Clinical data, imaging data, and molecular profiles from genomes, epigenomics, transcriptomics, proteomics, and metabolomics can all be part of these datasets (120).

DL and ML algorithms are excellent at finding small, non-linear relationships in big data sets. These models can detect early molecular markers and prediction patterns that are linked to the progression of oral squamous cell carcinoma (OSCC), even before there are any visible abnormalities in the tissue. AI systems can tell the difference between high-risk lesions and low-risk diseases more accurately than traditional methods by learning from high-dimensional data (119, 121).

AI-based models can use more than just genetic data. They can also use imaging methods, including histology slides, oral pictures, and X-rays. It is possible to automatically uncover dysplastic alterations, measure architectural difficulties, and find spatial heterogeneity inside lesions by using convolutional neural networks (CNNs) for advanced image processing. This combination of many modalities enhances diagnostic precision and facilitates objective, regular assessments of disease progression (122).

AI-based prediction models are important for personalized treatment since they let you figure out how risky something is for each person. These models can integrate patient data and predictive algorithms to generate tailored risk scores that assist physicians in determining the frequency of patient monitoring, the methodology for biopsies, and the optimal timing for initiating therapy. This technique transforms our approach to cancer treatment from a reactive stance to a proactive one, focusing on preventing its progression (122).

AI-based models hold significant potential; however, we must address numerous challenges before their clinical implementation. The challenge encompasses the requirement for extensive, well-annotated datasets, the uniformity of data-gathering methodologies, and the imperative to conduct testing across a diverse population (119, 122). Dealing with ethical, reproducibility, and interpretability issues is also essential to ensure the model’s trustworthiness and clinical application.

AI-driven predictive models represent a significant advancement in the management of OPMDs overall. These tools facilitate the early identification of issues, stratify individuals by risk, and deliver personalized treatment by transforming complex biological data into actionable insights. Ultimately, these advancements will enhance the efficacy of mouth cancer prevention (119).

Recent advances in single-cell sequencing and spatial transcriptomics have revealed more about the cellular heterogeneity and spatial organization of OPMDs and OSCC. These technologies enable characterization of epithelial, stromal, and immune cell populations at single-cell resolution while preserving tissue architecture, thereby improving understanding of tumor evolution, immune interactions, and microenvironmental remodeling during malignant transformation (117, 123).

Recent studies have further demonstrated the translational potential of integrated multi-omics and artificial intelligence-based approaches in OPMD and OSCC research. For example, salivary metabolomic profiling combined with microbiome analysis identified discriminatory metabolic signatures capable of differentiating OPMDs from OSCC, highlighting the utility of non-invasive biomarker discovery approaches (124, 125). In addition, salivary exosome-based multi-omics analyses have emerged as promising strategies for early diagnosis and prognosis, particularly through the characterization of exosomal miRNAs, proteins, and lipids involved in tumor progression and immune modulation (126). Furthermore, recent artificial intelligence and deep learning models have shown high diagnostic performance in the automated detection and classification of OPMDs and OSCC using histopathology and imaging datasets, supporting their potential application in precision diagnostics and risk stratification (127).

Despite the promising potential of biomarkers, multi-omics technologies, and artificial intelligence based diagnostic approaches, several challenges limit their current clinical translation. Most proposed biomarkers and AI models remain at the preclinical or early validation stage. Moreover, only a limited number of biomarker-driven and AI-assisted diagnostic approaches have undergone large-scale prospective clinical trial evaluation, and most available studies remain retrospective or based on small patient cohorts. This lack of robust clinical validation currently limits their integration into routine clinical practice and evidence-based decision-making.

In addition, regulatory approval, standardization of analytical platforms, reproducibility across populations, and data integration remain major barriers to routine clinical implementation. These challenges are particularly significant in low- and middle-income countries, where the burden of OPMDs and OSCC is highest but access to advanced molecular diagnostics and computational infrastructure remains limited. Therefore, the development of cost-effective, minimally invasive, and clinically validated diagnostic strategies suitable for resource-limited settings remains an important priority for improving early detection and precision oral cancer management.

## 9 Management strategies

The management of orally probable malignant diseases (OPMDs) and oral squamous cell carcinoma (OSCC) has evolved from a lesion-specific methodology to a dynamic, risk-stratified, and patient-centered framework. The primary principle of management is the swift recognition of progression and tumor stages in OPMDs and OSCC, respectively (3, 119). Management strategies require a blend of monitoring, adjustment of behavioral influences, lesion-targeted local therapy, and comprehensive treatment approaches, as these conditions arise from complex interactions involving carcinogenic exposure, genetic predispositions, immune evasion, and changes in the tissue microenvironment. Advancements in techniques such as surgical procedures, precision radiotherapy, molecular-guided systemic therapy, and immunotherapy have expanded the treatment alternatives for oral malignancies. This has resulted in a trade-off between tumor management and the preservation of function, quality of life, and long-term survival (3, 119, 128).

### 9.1 Surveillance, habit cessation, surgical excision, chemoprevention

**Figure 6:**
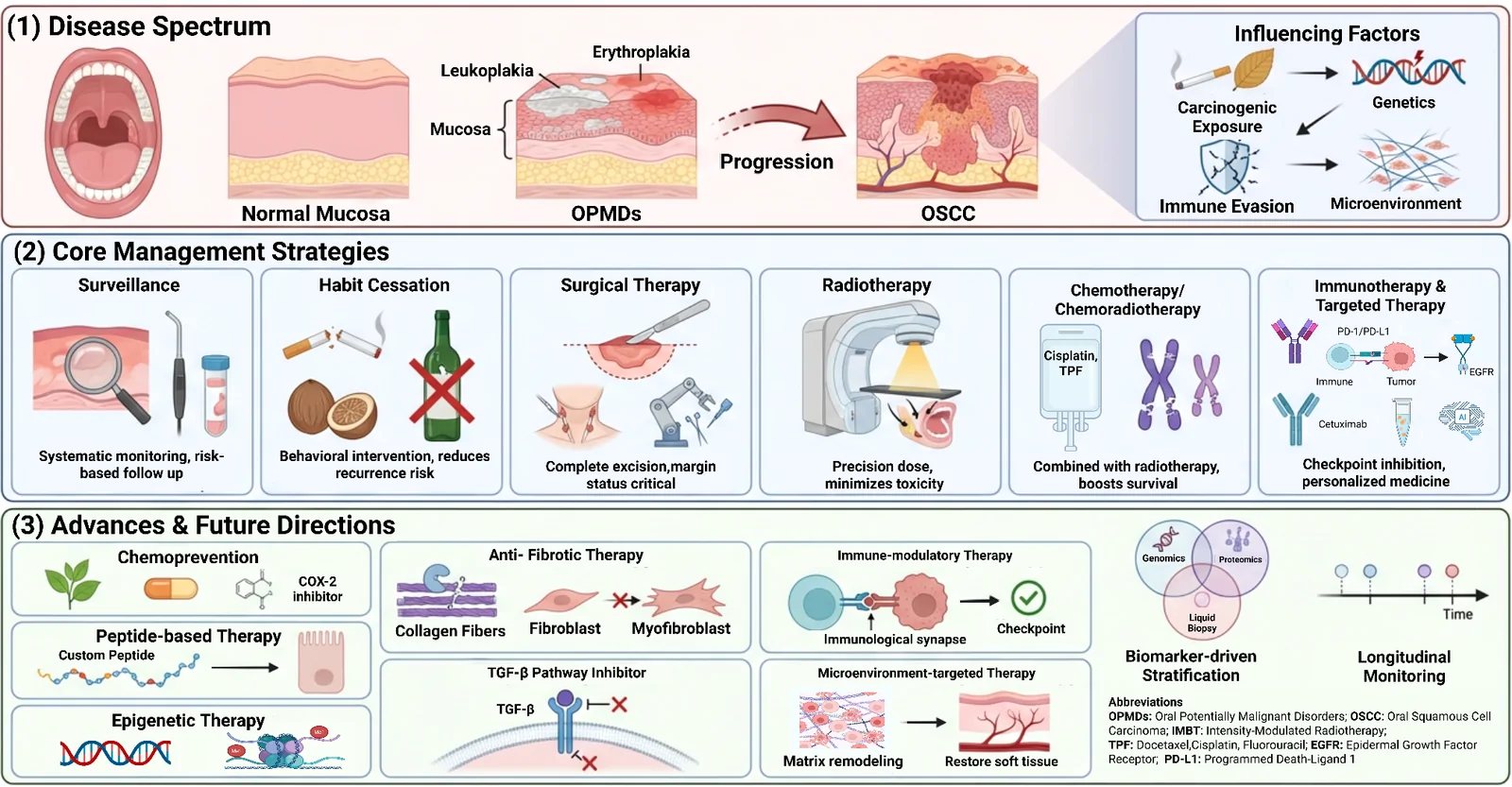
Multilevel framework of disease progression, therapeutic interventions, and future directions in OPMD-associated oral carcinogenesis.

Management of OPMDs relies on four interlinked strategies that include vigilant surveillance, cessation of carcinogenic habits, surgical excision, and chemoprevention for lesion control and prevention of malignant transformation (**Figure 6**). Although OPMDs present a quantifiable risk of advancing to invasive cancer, routine surveillance through comprehensive intraoral examination, photographic documentation, and periodic biopsy of atypical alterations is essential (129). The consensus guidelines stress about follow up intensity according to histological dysplasia grade, lesion size, lesion location, and patient risk profile.

Cessation of key risk habits like smoking, tobacco consumption, areca nut chewing, and excessive alcohol is non-negotiable. Habit cessation not only reduces the probability of new lesions but also slows down the progression of existing lesions. Studies on transtheoretical models reveal many patients with OPMDs remain in pre-contemplation stages with respect to tobacco cessation, underlying the need for behavioral intervention (130).

When histopathological analysis indicates moderate to severe dysplasia or clinical characteristics imply a high malignant potential, such as heterogeneous leukoplakia, increased size, and high-risk location, surgical removal is frequently advised. Surgical excision is optimal for high-risk lesions, stratified by the consensus guidelines. Although excision is the optimal management strategy, recurrence and malignant transformation may occur. Retinoids, COX-2 inhibitors, topical bleomycin, and photodynamic therapy are the chemotherapeutics that have been trialed against oral cancer; however, results are inconsistent, and toxicity remains a drawback (131). A systematic analysis reported a mean full response rate of approximately 32% for topical retinoid therapy in oral potentially malignant disorders (OPMDs). In the future, chemopreventive research needs to identify high-risk subsets and deploy agents with favorable safe profiles.

### 9.2 Surgery treatment

Surgery remains the most effective treatment for OSCC, especially in early-stage diseases and resectable advanced tumors. The primary goal of surgery is the complete excision of the tumor with histologically negative margins while retaining the oral function and facial aesthetics (131, 132). Margin status is a well-known prognostic factor in OSCC, where positive or close margins (<5 mm) are significantly associated with local recurrence and poor survival outcomes. Generally, doctors recommend wide local excision with a minimum of a 5 mm tumor-free margin (**Figure 6**).

Wide local excision with a minimum of a 5 mm tumor-free margin is commonly adopted in clinical practice, although several recent studies have demonstrated that margin adequacy must be viewed in the context of tumor biology and depth of invasion (DOI) (133). The 8th edition of the AJCC staging system recognizes depth of invasion as a key prognostic factor of tumor staging and surgical planning, as increased DOI is closely linked to nodal metastasis and reduced disease-specific survival.

Cervical lymph node metastasis is still the most important prognostic factor when evaluating the degrees of cancer spread (staging) in oral squamous cell carcinoma (OSCC). Even in patients who are clinically node-negative (cN0), the rates of occult metastasis are between 20 and 30 percent, which is why neck dissections are justified (133, 134). For early-stage disease, selective neck dissections (levels I-III) are the standard of care, but for clinically node-positive disease, modified radical or comprehensive neck dissections are required. At advanced stages of the disease, sentinel lymph node biopsy is considered a better standard of care than elective neck dissections because it is less invasive. This approach has been proven in studies with multiple centers. There are still some barriers, including the availability of resources and the required skills to make it routine (134).

Some minimally invasive methods, such as transoral laser microsurgery (TLM) and transoral robotic surgery (TORS), have been accepted for some cases of OSCC (oral squamous cell carcinoma), particularly for oropharyngeal and tongue tumors (135). These methods provide better visual access and have been correlated with less operative morbidity and better functional outcomes. Reconstructive surgery has been revolutionized by the incorporation of microvascular free flaps. These treatments have established themselves as the gold standard for restoring form and function following most difficult resections. Fibula free flaps are the most often utilized method for mandibular repair. Moreover, radial forearm and anterolateral thigh free flaps are frequently employed for soft tissue abnormalities (136).

### 9.3 Radiotherapy in OSCC

Radiotherapy is a basic element of OSCC treatment which we use as a primary, adjuvant, or palliative tool based on disease stage and patient factors. Researchers highly advocate for postoperative irradiation in patients exhibiting adverse pathological characteristics, including positive margins, extracapsular nodal extension (ENE), perineural invasion, lymphovascular invasion, and advanced T or N stage (133). Definitive radiotherapy may be considered for patients that are beyond the means of surgery or who decline it, but in general local control is not as effective as what is seen with surgery-based multimodality treatment. The introduction of intensity modulated radiotherapy (IMRT) has transformed OSCC treatment by enabling a very precise dose to the tumor at the same time, critical structures like salivary glands and the mandible are protected (133, 137).

IMRT has been proven to cut xerostomia significantly and enhance the quality of life without endangering oncological results. Volumetric modulated arc therapy (VMAT) and image-guided radiotherapy (IGRT) double the accuracy of treatment and cut the time taken for treatment (**Figure 6**) (133). Proton therapy is a new approach that has potential dosimetric advantages, however, there is still a lack of clinical evidence in OSCC. Despite the cutting-edge technology, radiotherapy-induced toxicities, including mucositis, dysphagia, osteoradionecrosis, and trismus are still very challenging. Tumor hypoxia and intrinsic radioresistance are the main factors that make treatment fail in a group of patients (138).

### 9.4 Chemotherapy and chemoradiotherapy

Supportive but significant chemotherapy is a major part of the OSCC treatment plan and is mainly used in conjunction with radiotherapy for advanced and high-risk postoperative cases. The concurrent chemoradiotherapy (CCRT) using cisplatin-based regimens is acknowledged as the standard treatment for patients with positive margins or ENE after surgery (139). Comparative studies have shown a consistent enhancement of CCRT in terms of locoregional control and overall survival when compared to radiotherapy alone but, at the same time, with a higher incidence of acute and late toxicities (**Figure 6**).

Induction chemotherapy (ICT) is the process of shrinking the tumor and killing the microscopic disease that cannot be seen before the definitive treatment. The TPF regimen (docetaxel, cisplatin, and 5-fluorouracil) has had a better response rate compared to PF regimens. On the other hand, large-scale randomized studies have not consistently been able to show that ICT leads to better survival comparable to standard CCRT, which restricts its application in routine selected high-risk or borderline cases of resectable tumors. The combination of platinum-based chemotherapy with 5-fluorouracil and cetuximab was considered standard first-line treatment for recurrent or metastatic OSCC (EXTREME regimen) for a long time (140). However, the outcomes remained inadequate, ultimately prompting the exploration of novel remedies utilizing immunotherapy methods.

### 9.5 Immunotherapy

Immunotherapy is a new treatment method for recurrent and metastatic OSCC that has profoundly changed the treatment landscape. Immune checkpoint inhibitors (ICIs) acting on the programmed death-1 (PD-1) receptor and its ligand PD-L1 have shown consistent responses and even increased survival. The CheckMate-141 trial positioned nivolumab as the standard treatment for platinum-refractory head and neck squamous cell carcinoma, showing a remarkable overall survival benefit over standard chemotherapy (140, 141). The KEYNOTE-048 trial demonstrated the superiority of pembrolizumab, both as monotherapy and in conjunction with chemotherapy, in PD-L1-positive recurrent or metastatic illness (**Figure 6**).

Among the various biomarkers for immunotherapy selection, PD-L1 expression, determined by the combined positive score (CPS), is the most commonly used. A higher CPS correlates with better response and survival outcomes (142). Tumor mutational burden, immune gene signatures, and microbiome composition are potential predictive markers under study. Only 15–25% of the patients are able to get significant responses to ICIs even though the results are promising. Immune-related adverse events, treatment resistance, and the absence of reliable biomarkers are the hurdles to be overcome. Researchers are currently conducting studies to assess combination strategies that integrate immunotherapy with radiotherapy, chemotherapy, and targeted agents (143).

### 9.6 Targeted therapy

The aim of targeted therapies is to stop specific molecular pathways which contribute to OSCC development. The epidermal growth factor receptor (EGFR) exists in more than 80% of OSCC cases, and its presence predicts both aggressive tumor behavior and poor patient outcomes. Cetuximab represents the main targeted treatment for OSCC research because it acts as an anti-EGFR monoclonal antibody (144). The treatment shows better locoregional control when combined with radiotherapy for patients who have locally advanced disease. The research indicated that cetuximab performed worse than cisplatin-based CCRT after researchers conducted their comparison of both treatments (**Figure 6**).

The researchers employed next-generation sequencing to find changes in the TP53, PIK3CA, NOTCH, and CDKN2A genes in OSCC. The current efforts to achieve successful results through the PI3K/AKT/mTOR pathway and angiogenesis signaling mechanisms remain unsuccessful, while immunotherapy combinations demonstrate potential. The future of OSCC treatment will use personalized therapies, which depend on specific biomarker information. The new liquid biopsy approaches, which detect circulating tumor DNA and exosomal biomarkers, provide an effective solution for early cancer recurrence detection and treatment response evaluation (144, 145). Artificial intelligence-based imaging and molecular profiling methods will improve the process of determining treatment selection and predicting patient outcomes.

## 10 Therapeutic interception and prevention

The progression of oral potentially malignant disorders (OPMDs) necessitates a shift from treating advanced malignancies to implementing early preventive strategies. Intervening at the premalignant stage provides a crucial opportunity to halt cancer development, as these lesions possess biological plasticity that can undergo therapeutic modulation. Chemoprevention focuses on targeting early molecular drivers such as chronic inflammation, oxidative stress, epithelial plasticity, and microenvironmental remodeling (146). Natural compounds, particularly phytochemicals, have gained attention due to their antioxidant and anti-inflammatory properties, which help neutralize reactive oxygen species, suppress pro-tumorigenic signaling, and restore redox balance. Peptide-based therapies provide high specificity in modulating signaling pathways involved in proliferation, apoptosis, and immune regulation, thereby stabilizing epithelial differentiation and limiting invasive potential. Targeted molecular therapies further enhance prevention by selectively inhibiting dysregulated pathways governing cell cycle progression and survival while maintaining favorable safety profiles (69).

Epigenetic therapies exploit the reversible nature of early molecular alterations, enabling restoration of normal gene expression, reactivation of tumor suppressor genes, and reduction of stem-like characteristics. Immunomodulatory approaches aim to counteract immune evasion and tolerance, enhancing immune surveillance to eliminate high-risk cells before malignant transformation (146). Additionally, strategies targeting the tumor microenvironment such as modulation of stromal activity, extracellular matrix remodeling, inflammation, and angiogenesis help disrupt the supportive niche required for cancer progression. Effective therapeutic interception relies on integrating molecular profiling, biomarker-driven risk stratification, and longitudinal monitoring to guide personalized interventions (147).

Overall, combination approaches targeting multiple interconnected pathways are likely to be more effective than single-agent therapies. Rather than solely inducing lesion regression, the goal is to achieve durable reprogramming toward a non-malignant state. This preventive, biology-driven strategy redefines OPMD management, offering a promising pathway toward sustained disease control and the prevention of oral cancer development.

### 10.1 Anti-fibrotic therapies

Anti-fibrotic techniques are a crucial method for halting the early evolution of OSMF by addressing the abnormal buildup of extracellular matrix and the rigidity of tissues. Changes caused by fibrosis, such as too much collagen production, low oxygen levels, and poor blood flow, generate a milieu that encourages epithelial change (17, 147). Therapeutic strategies targeting the reduction of collagen synthesis, the enhancement of matrix breakdown, or the inhibition of fibroblast-to-myofibroblast development can facilitate the restoration of tissue homeostasis. Anti-fibrotic medicines may inhibit mechanotransduction-driven signaling and impede the advancement towards malignancy by altering the fibrotic microenvironment.

### 10.2 TGF-β pathway inhibitors

Targeting the transforming growth factor-β (TGF-β) pathway is a potential therapeutic approach, as it is integral to fibrosis, epithelial–mesenchymal transition, and immune suppression. TGF-β signaling regulates the deposition of the extracellular matrix, the activation of myofibroblasts, and the plasticity of epithelial cells (**Figure 6**) (148). All of these variables contribute to the growth of OSMF and its malignancy. Pharmacological suppression of TGF-β signaling, encompassing receptor kinase inhibitors and downstream pathway modulators, has been useful in diminishing fibrotic activity and tumor-promoting effects. Nonetheless, selective targeting is essential due to the bifunctional role of TGF-β in maintaining tissue homeostasis and modulating immune responses.

### 10.3 Epigenetic therapy

Epigenetic therapies seek to rectify abnormal gene expression patterns that facilitate OPMD progression and malignant transformation. A major cause of tumor suppressor genes being turned off and oncogenic pathways being turned on is the dysregulation of DNA methylation, histone changes, and non-coding RNAs (**Figure 6**) (148, 149). Agents that target these reversible changes could bring back normal transcriptional programs and stop the disease from getting worse. Epigenetic therapy enhances cellular sensitivity to further therapies, rendering it a valuable component of combination therapy strategies.

### 10.4 Immunotherapy strategies

Epigenetic therapies aim to correct aberrant gene expression patterns that promote the evolution of OPMD and malignant transformation. The dysregulation of DNA methylation, histone modifications, and non-coding RNAs is a primary factor in the inactivation of tumor suppressor genes and the activation of carcinogenic pathways (**Figure 6**) (150). Agents that target these reversible alterations could restore normal transcriptional pathways and prevent disease progression. Epigenetic therapy can also make cells more responsive to other treatments, which makes it a good addition to combination therapy strategies.

## 11 Challenges and future directions

Despite increasing knowledge about the molecular and cellular processes that cause oral potentially malignant disorders (OPMDs), their clinical treatment faces multiple obstacles which remain unsolved. Researchers face their greatest obstacle when they try to study how OPMDs progress over time because they lack access to ongoing research studies which monitor patients from their first symptoms until they reach their potentially malignant stage (4). Most research studies at present use cross-sectional methods, which provide researchers with single molecular snapshots that do not show how premalignant lesions change and adapt over time. The absence of longitudinal monitoring makes it impossible to detect early signals which predict future events and track how particular cell lineages change and identify the order through which genetic and epigenetic and environmental factors drive disease progression (151).

The second primary challenge exists because diagnostic criteria suffer from both variability and subjective interpretation, especially during histopathological evaluations. The grading process for epithelial dysplasia assessment suffers from problems because observers show different results when they evaluate the same cases, and actual results show that similar-looking lesions produce distinct clinical outcomes (152). The existing procedures create problems for risk assessment because they prevent accurate prediction through morphological assessment. The traditional diagnostic method fails to recognize field cancerization together with molecular changes which occur in histologically normal-appearing mucosa, leading to incorrect assessments of malignant risk for specific patients.

Current clinical practices do not incorporate molecular biomarkers in their standard testing procedures. Research has discovered many potential biomarkers, but only a small selection has passed comprehensive testing across various population groups and healthcare conditions. The implementation of omics-based diagnostics faces three primary hurdles, which include the absence of standardized sampling procedures and the expensive nature of molecular tests, and the low level of doctor expertise in these diagnostic methods. Most biomarker research studies investigate only single molecules, which leads to an incomplete understanding of OPMD biological systems (153). The clinical implementation process becomes slower because predictive models build through different research methods, which create fragmented information.

Systems biology methods need to shift from current systems toward their systems biology methods, which combine genomic data with epigenomic data and transcriptomic data and proteomic data and metabolomic data and microenvironmental data to build unified disease progression models (154). Malignant transformation arises from dysregulation at the network level, necessitating several genetic alterations. Researchers can use computational modeling and artificial intelligence to analyze complex datasets, which enables them to discover hidden patterns that create superior predictive models compared to conventional risk assessment methods.

Researchers need to investigate two important research areas, which include longitudinal research and minimally invasive research needs. Researchers can collect saliva and blood and exfoliate cells through repeated testing, which enables them to observe molecular changes that occur during the development of precancerous conditions. The dynamic assessment approach enables researchers to observe how changes occur over time, which helps them detect disease progression and regression patterns for developing early intervention methods and adaptive management plans (154, 155). The combination of longitudinal sampling methods with patient outcome data will help researchers establish biomarker validation studies while developing advanced predictive modeling methods.

The field requires translational studies which establish direct links between molecular changes and clinical decision processes. Future research should investigate the impact of molecular data on patient surveillance schedules, treatment methodologies, and medical guidance, rather than treating biomarkers as discrete entities (156). Molecular profiling should be incorporated into clinical trials to enhance scientific validation and promote the implementation of research findings in practical contexts.

Research studies must investigate the influence of diverse demographics and environmental factors on their outcomes. Three factors influence the biological characteristics and clinical consequences of OPMD conditions: genetic variety, diverse daily lifestyles, and access to healthcare services (156, 157). Multisite research initiatives will enhance the creation of predictive models for devising treatment methods customized for various patient demographics.

The future of OPMD research requires researchers to study complicated systems instead of trying to make them simpler. Researchers can get precise forecasts and preventive strategies by integrating longitudinal data with systems-level analysis and translational frameworks (157). Interdisciplinary cooperation will assist researchers in addressing current challenges, facilitating the transformation of OPMDs from erratic precursors into manageable and treatable phases of oral carcinogenesis.

## 12 Current challenges and unresolved questions

Despite significant advances in understanding OPMD-to-OSCC progression, several important controversies and unresolved questions remain. Histopathological grading continues to show limited predictive accuracy due to substantial interobserver variability and inconsistent correlation with malignant transformation risk (158). In addition, considerable heterogeneity exists among different OPMDs regarding molecular alterations, clinical behavior, and transformation potential, making universal risk prediction challenging. Although multiple biomarkers and signaling pathways have been proposed, their clinical utility and reproducibility remain insufficiently validated across large longitudinal cohorts (158, 159). Furthermore, the complex interactions among epithelial cells, stromal components, immune cells, and extracellular matrix remodeling within the tumor microenvironment are not yet fully understood. The dynamic crosstalk between signaling pathways and stage-specific molecular alterations also remains an area of active investigation. Future studies integrating longitudinal multi-omics profiling, spatial transcriptomics, and artificial intelligence–based predictive modeling may help address these challenges and improve precision risk stratification and targeted prevention strategies (160).

## 13 Conclusion

Oral potentially malignant disorders (OPMDs) are dynamic precursor states that progress to oral squamous cell carcinoma (OSCC) through the interplay of molecular alterations and tumor microenvironment remodeling. Dysregulation of key signaling pathways, including TGF-β, PI3K/AKT, and Wnt/β-catenin, along with chronic inflammation and fibrosis, drives epithelial plasticity and OSCC development. Oral submucous fibrosis further highlights the role of extracellular matrix stiffness and mechanotransduction pathways such as YAP/TAZ in promoting malignant transformation. Emerging multi-omics–based biomarkers and artificial intelligence–driven models provide promising tools for early detection and risk stratification of OSCC. A systems-level understanding of OPMD progression is essential for advancing precision oncology, enabling improved early diagnosis, targeted prevention, and better clinical outcomes in OSCC.
