# Correction: Molecular and microenvironmental drivers of malignant transformation from oral potentially malignant disorders to oral squamous cell carcinoma

**DOI:** 10.3389/fonc.2026.1950779

**Published:** 2026-08-11

**Authors:** Vasundra V., Shajitha R., Sundaresan S., Magesh R.

**Affiliations:** 1 Department of Biotechnology, Faculty of Biomedical Sciences & Technology, Sri Ramachandra Institute of Higher Education and Research (DU), Chennai, India; 2 Division of Medical Research, SRM MCH and RC, SRM IST, Kattankulathur, Tamil Nadu, India

**Keywords:** biomarkers, healthcare, malignant transformation, multi-omics, oral potentially malignant disorders, oral squamous cell carcinoma, precision oncology, tumor microenvironment

The funder “SRM Institute of Science and Technology, Kattankulathur” to “Sundaresan S” was erroneously omitted. The correct **Funding** statement reads:

“The author(s) declared that financial support was received for this work and/or its publication. This work was funded by the SRM Institute of Science and Technology, Kattankulathur, Tamil Nādu, India- Open access Publication Fund”.

Affiliation 2 “Division of Medical Research, SRM MCH and RC, SRM IST, Kattankulathur, Tamil Nadu, India” was listed incorrectly for author “Sundaresan S”.

The city of affiliation 2 was erroneously written as “Chennai”. Affiliation 2 has been corrected to “Division of Medical Research, SRM MCH and RC, SRM IST, Kattankulathur, Tamil Nadu, India”.

The original version of this article has been updated.

